# Investigating Multi-Omic Signatures of Ethnicity and Dysglycaemia in Asian Chinese and European Caucasian Adults: Cross-Sectional Analysis of the TOFI_Asia Study at 4-Year Follow-Up

**DOI:** 10.3390/metabo15080522

**Published:** 2025-08-01

**Authors:** Saif Faraj, Aidan Joblin-Mills, Ivana R. Sequeira-Bisson, Kok Hong Leiu, Tommy Tung, Jessica A. Wallbank, Karl Fraser, Jennifer L. Miles-Chan, Sally D. Poppitt, Michael W. Taylor

**Affiliations:** 1 School of Biological Sciences, University of Auckland, Auckland 1010, New Zealand; sfar315@aucklanduni.ac.nz (S.F.); i.sequeira@auckland.ac.nz (I.R.S.-B.); klei603@aucklanduni.ac.nz (K.H.L.); htun832@aucklanduni.ac.nz (T.T.); jwal670@aucklanduni.ac.nz (J.A.W.); s.poppitt@auckland.ac.nz (S.D.P.); 2 Human Nutrition Unit, University of Auckland, Auckland 1024, New Zealand; 3 High-Value Nutrition National Science Challenge, Auckland 1023, New Zealand; ajoblinmills@gmail.com (A.J.-M.); karl.fraser@agresearch.co.nz (K.F.); 4 AgResearch Limited, Palmerston North 4442, New Zealand; 5 Riddet Institute, Massey University, Palmerston North 4442, New Zealand; 6 Department of Medicine, University of Auckland, Auckland 1023, New Zealand

**Keywords:** multi-omics, shotgun metagenomics, plasma metabolomics, ethnicity, glycaemic status, gut microbiome, type 2 diabetes, triglycerides, BCAA, *Phocaeicola vulgatus*, host–microbiome interactions

## Abstract

**Background:** Type 2 diabetes (T2D) is a global health epidemic with rising prevalence within Asian populations, particularly amongst individuals with high visceral adiposity and ectopic organ fat, the so-called Thin-Outside, Fat-Inside phenotype. Metabolomic and microbiome shifts may herald T2D onset, presenting potential biomarkers and mechanistic insight into metabolic dysregulation. However, multi-omics datasets across ethnicities remain limited. **Methods:** We performed cross-sectional multi-omics analyses on 171 adults (99 Asian Chinese, 72 European Caucasian) from the New Zealand-based TOFI_Asia cohort at 4-years follow-up. Paired plasma and faecal samples were analysed using untargeted metabolomic profiling (polar/lipid fractions) and shotgun metagenomic sequencing, respectively. Sparse multi-block partial least squares regression and discriminant analysis (DIABLO) unveiled signatures associated with ethnicity, glycaemic status, and sex. **Results:** Ethnicity-based DIABLO modelling achieved a balanced error rate of 0.22, correctly classifying 76.54% of test samples. Polar metabolites had the highest discriminatory power (AUC = 0.96), with trigonelline enriched in European Caucasians and carnitine in Asian Chinese. Lipid profiles highlighted ethnicity-specific signatures: Asian Chinese showed enrichment of polyunsaturated triglycerides (TG.16:0_18:2_22:6, TG.18:1_18:2_22:6) and ether-linked phospholipids, while European Caucasians exhibited higher levels of saturated species (TG.16:0_16:0_14:1, TG.15:0_15:0_17:1). The bacteria *Bifidobacterium pseudocatenulatum*, *Erysipelatoclostridium ramosum,* and *Enterocloster bolteae* characterised Asian Chinese participants, while *Oscillibacter* sp. and *Clostridium innocuum* characterised European Caucasians. Cross-omic correlations highlighted negative correlations of *Phocaeicola vulgatus* with amino acids (r = −0.84 to −0.76), while *E. ramosum* and *C. innocuum* positively correlated with long-chain triglycerides (r = 0.55–0.62). **Conclusions**: Ethnicity drove robust multi-omic differentiation, revealing distinctive metabolic and microbial profiles potentially underlying the differential T2D risk between Asian Chinese and European Caucasians.

## 1. Introduction

Diabetes and its associated comorbidities are a major global health concern, with >500 million people currently living with type 2 diabetes (T2D), projected to rise to ~800 million by 2045 [1]. Type 2 diabetes is a disease characterised by the dysregulation of protein, lipid, and carbohydrate metabolism and accounts for >90% of all diabetes cases [2]. Prediabetes precedes T2D and is identified by impaired fasting glucose (IFG), impaired glucose tolerance (IGT), and/or increased glycated haemoglobin A_1c_ (HbA_1c_) [2]. Prediabetes can rapidly develop into frank T2D in affected individuals, occurring at an annual estimated rate of 3–11% [2]. As a critical intermediate glucoregulatory stage, prediabetes thus offers a window of opportunity, during which interventions may prevent progression to T2D. Prediabetes is estimated to affect >850 million individuals worldwide, forecast to rise to ~1.2 billion by 2045 [1].

T2D is becoming increasingly prevalent among Asian people, with a particularly concerning rise among young adults and children [3,4]. China, for example, has experienced a dramatic increase in the prevalence of adult obesity, increasing from ~30% in 2002 to ~51% in 2015–2019 [5,6]. Adiposity underpins the pathogenesis of T2D, with people of Asian heritage tending to have a higher percentage of total body and abdominal fat and lower lean mass compared to European counterparts with a similar body mass index (BMI) [4,7]. This ethnicity-dependent differential in fat partitioning and body composition [8] reflects the TOFI (Thin-on-the-Outside, Fat-on-the-Inside) phenotype described originally by Thomas and colleagues [9]. The TOFI profile is characterised by high abdominal and visceral adipose tissue (VAT) distribution in outwardly thin individuals [9] and differs between ethnic groups [8]. Chinese individuals with the TOFI phenotype accumulate VAT in response to modest weight gain. This results in ectopic fat infiltration in critical organs such as the liver and pancreas, promoting hepatic insulin resistance and β-cell dysfunction [10,11,12].

The human gut microbiome has received considerable attention due to its profound contribution to human health and the therapeutic potential offered by its inherent manipulability [13,14]. Gut microbes play a crucial role in maintaining host homeostasis and, thus, host health by regulating energy balance [15], modulating the immune system [16], and via competitive exclusion of pathogens [17,18]. The gut microbiome ferments indigestible complex dietary components, generating microbial metabolites such as short-chain fatty acids (SCFAs) acetate, propionate, and butyrate [16]. These SCFAs contribute to host metabolism and can also act as metabolic modulators, with evidence that butyrate enhances insulin sensitivity and induces glycolysis [19], while acetate improves glucose homeostasis [20]. Metagenome-wide association studies of Chinese individuals with T2D have shown moderate microbiome dysbiosis, including decreases in the abundance of butyrate-producing bacteria and increases in various opportunistic pathogens [21]. Compositional and functional characteristics of the faecal microbiome have been associated with T2D status in European individuals [22]. When re-analysed with the Chinese study above [21], discriminant metagenomic markers for T2D differed between the Chinese and European cohorts [22]. Significant differences between the faecal microbiomes of European- and Chinese-ancestry cohorts with overweight and prediabetes living in New Zealand were also observed in a recent study from our laboratory, using 16S rRNA gene-based methods, from which the underpinning factors could not be determined [23]. Finally, the longitudinal China Health and Nutrition Survey, which included 2772 Chinese participants, established that members of the gut microbiome linked with improved glycaemic traits were also associated with healthier dietary habits, such as higher intakes of vegetables, fruits, fish, and nuts [24].

In parallel with microbiome differences between Chinese and European cohorts, recent plasma metabolomic (lipids and polar metabolites) analyses of baseline data from our New Zealand-based TOFI_Asia study identified distinct metabolic features for visceral adiposity and dysglycaemia between Chinese and European adults [25]. FPG was significantly correlated with more and different metabolites in Chinese individuals than in their European counterparts (110 vs. 40 metabolites, respectively), as was the ratio of visceral adiposity to total body fat [25]. The potential for lipids and polar metabolite features to act as early biomarkers of prediabetes were shown in both ethnicities through higher total and central adiposity, adverse clinical markers, and liver enzymes, and higher glucoregulatory peptides in individuals with normal FPG but a prediabetes metabolomic profile [25].

Building upon our previous baseline TOFI_Asia findings [25,26] from 2016 to 2017, we performed a cross-sectional analysis of samples collected 4 years later (~2020–2021) in the TOFI_Asia follow-up study. This snapshot analysis utilises a multi-omics approach consisting of shotgun faecal metagenomics and plasma metabolomics (lipids and polar metabolites) to construct a more comprehensive and robust analysis of microbial and metabolic features. To leverage the complexity of this dataset, we applied multivariate modelling tools from the mixOmics framework, including sparse Partial Least Squares Discriminant Analysis (sPLS-DA) and DIABLO (Data Integration Analysis for Biomarker discovery using Latent Components). These methods allowed for feature selection and integration across omic datasets while maintaining a biologically meaningful structure, providing insight into discriminant and correlated biological signals [27,28]. Sex hormones can directly regulate metabolic enzymes such as fatty acid synthase, lipases, and glycolytic enzymes, resulting in sexually dimorphous metabolic profiles that may underlie disease susceptibility between males and females [29,30,31,32]. Characterising sex-specific metabolic signatures should facilitate biomarker discovery and precision medicine development, as metabolite profiles often exhibit sex-dependent correlations with metabolic diseases [33,34]. We applied this approach to samples obtained in the TOFI_Asia Follow-Up study from a New Zealand cohort of 99 Asian Chinese and 72 European Caucasian adults with varying body weight, adiposity, and glycaemic status, thus yielding insights into the interplay between ethnicity and glycaemic regulation in a metabolically at-risk population. The aim of this study was thus to perform a cross-sectional multi-omics analysis of the TOFI_Asia cohort at a 4-year follow-up, integrating both faecal metagenomics and plasma metabolomics to identify ethnicity- and glycaemia-associated signatures and cross-omic correlations in Asian Chinese and European Caucasians.

## 2. Materials and Methods

### 2.1. Study Background

The baseline TOFI_Asia study was conducted at the University of Auckland’s Human Nutrition Unit under the auspices of the High-Value Nutrition National Science Challenge. Participants self-reported as being of either Asian Chinese or European Caucasian ethnicity. The recruitment age for both sexes ranged from 20 to 70 years, BMI (20–45 kg/m^2^), and glycaemic state (normoglycaemia or prediabetes identified on the basis of fasting plasma glucose (prediabetes FPG: 5.6–6.9 mmol/L), according to American Diabetes Association guidelines [35]). Exclusion criteria included weight gain or loss greater than 10% within the last three months, bariatric surgery, pregnancy, breastfeeding, use of glucose-related medications, or a current history of metabolic disease, including T2D. A more detailed description of the TOFI_Asia study protocol and cohort has been published elsewhere [26]. In total, 357 participants were recruited for the baseline TOFI_Asia study: 199 of Asian Chinese descent and 158 of European Caucasian descent [25,26].

**Figure 1:**
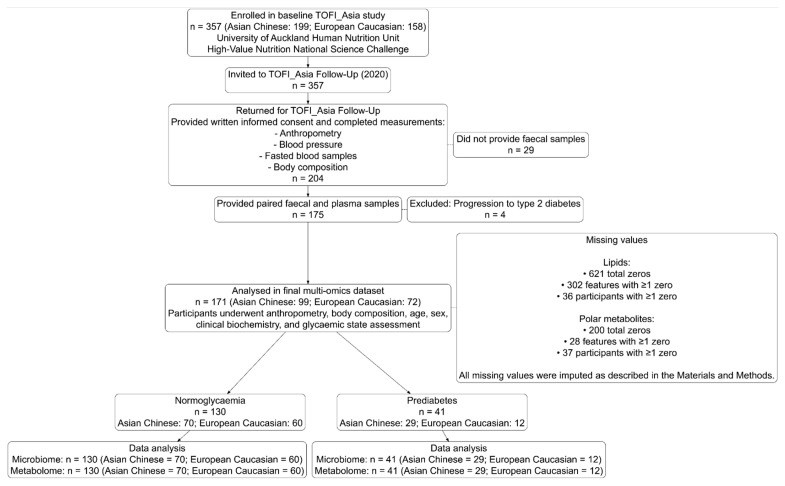
Participant flowchart for the faecal metagenomics and plasma metabolomics analyses.

In 2020, 204 participants returned for the TOFI_Asia Follow-Up study (Figure 1). Of the 204, 175 provided both faecal and plasma paired samples at an average follow-up period of 4 years. Participants also had anthropometric measurements, body composition, age, sex, and clinical biometrics recorded (Table 1). Four participants were excluded due to progression from hyperglycaemia to frank T2D, resulting in a final multi-omics dataset of 171 participants. The final cohort comprised 99 Asian Chinese (40:59 M:F) and 72 European Caucasian (30:42), of whom 130 exhibited normoglycaemia (43:87 M:F; 70 Asian Chinese, 60 European Caucasian), and 41 had prediabetes (27:14 M:F; 29 Asian Chinese, 12 European Caucasian).

#### Ethics Approval and Trial Registration

The study was approved by the Southern Health and Disabilities Committee (HDEC), New Zealand (approval code: 16/STH/23/AMO9, approved 11 December 2019). The study was registered with the Australian New Zealand Clinical Trial Registry (ACTRN12621000001897, registered 7 January 2021). All participants provided written, informed consent prior to participation.

### 2.2. Sample Collection

#### 2.2.1. Faecal Sample Collection

Participants were provided with a sterile sample collection kit consisting of a kidney dish, a scoop, a container, and a sterile collection tube. Prior to sample collection, participants were instructed to maintain sterile conditions throughout the collection and storage processes, including avoiding contact between the collection material and non-sterile surfaces. Participants collected faecal samples at home utilising a sterile technique. Using the kidney dish and scoop, participants transferred faecal material into the sterile collection tube, which was sealed and placed into the transport container prefilled with water and frozen to create an insulating ice jacket. Samples were stored in participant home freezers at −18 °C for 24–48 h, prior to transport to the Human Nutrition Unit.

Samples remained in the insulated container, with the frozen water jacket, to minimise thawing during transit to the University of Auckland, where they were stored at −80 °C until the DNA extraction protocol was performed.

#### 2.2.2. Anthropometric, Clinical, and Biochemical Assessments

Anthropometric and clinical assessments were conducted at the Human Nutrition Unit (HNU), University of Auckland. Measurements included height, weight, waist and hip circumference, blood pressure, and fasting venous blood markers. Standardised protocols were adhered to in order to ensure accuracy and consistency.

Participants were assessed barefoot and lightly clothed. Height was measured to the nearest 0.1 cm using a wall-mounted stadiometer (Seca 222, Seca GmbH & Co., KG, Hamburg, Germany), and weight was measured to the nearest 0.1 kg using a digital scale (Mettler Toledo Spider, Mettler-Toledo International Inc., Greifensee, Switzerland). Body Mass Index (BMI) was calculated and categorised per WHO criteria. Waist circumference was measured at the midpoint between the lowest palpable rib and the iliac crest, and hip circumference was measured at the widest point over the greater trochanter; both measurements were recorded using a flexible anthropometric tape (Abbot Laboratories, IL, USA). Measurements were taken in duplicate and averaged.

Blood pressure was measured on the non-dominant arm using a calibrated electronic sphygmomanometer (Critikon DINAMAP^®^, GE Healthcare, Shanghai, China), with participants resting and seated. Two measurements were recorded two minutes apart, and the analysis used the average.

Dual-energy X-ray absorptiometry (DXA) was used to assess total and regional body composition, including adipose and lean tissue mass. Scans were conducted using the Lunar iDXA system (GE Healthcare, Madison, WI, USA) at the University of Auckland’s Clinical Research Centre. Participants were excluded if pregnant, ≥150 kg, or they had metal implants of any kind. Before the scan, participants removed footwear and metallic objects and lay supine on the scanner table with limbs positioned to avoid overlap. Each scan took 10 min. Body fat percentages, such as abdominal adipose tissue (AAT), subcutaneous adipose tissue (SAT), and visceral adipose tissue (VAT), were calculated as previously detailed by Sequeira et al. (2020) [26].

Fasting venous blood samples were collected at the HNU via single venipuncture or cannulation in the antecubital fossa of the participant’s arm. Blood was transferred into BD Vacutainer^®^ tubes (Becton Dickinson, Franklin Lakes, NJ, USA). Blood for glycated haemoglobin (HbA1c) analysis was collected in K3-EDTA tubes; plasma for glucose in fluoride oxalate tubes; serum for lipid and liver enzyme analyses in SST™ II advance gel separator tubes with clot activator; and plasma for glucoregulatory peptide analysis in P800 tubes containing spray-dried K2-EDTA and proprietary protease, esterase, and DDP-IV inhibitors to preserve peptide integrity. Immediately post-collection, tubes were gently inverted 6 to 8 times to prevent microclotting. All tubes were left at room temperature for 30–60 min, except for the p800 tubes, which were refrigerated at 4 °C per the manufacturer’s instructions. Samples were centrifuged at 3000 rpm for 10 min at 4 °C using an Eppendorf 5702R centrifuge (Eppendorf, Hamburg, Germany). The resulting plasma and serum were aliquoted into microcentrifuge tubes (EP0030120094, Eppendorf, Hamburg, Germany) and stored at −20 °C at the HNU, then transferred to −80 °C for long-term storage at the School of Biological Sciences, University of Auckland.

Plasma glucose was quantified using the hexokinase enzymatic method. Liver enzymes, including alanine aminotransferase (ALT) and aspartate aminotransferase (ALT), were analysed following the IFCC (International Federation of Clinical Chemistry) guidelines; alkaline phosphatase (ALP) via an IFF colourimetric assay; and gamma-glutamyl (GGT) transferase using the Szasz method.

Serum lipid concentrations such as total cholesterol, triglycerides, and high-density lipoprotein cholesterol (HDL-C) were measured enzymatically using clinical chemistry assays: total cholesterol by the cholesterol esterase/oxidase/peroxidase method; triglycerides by the lipase/glycerol kinase method; and HDL-C using a detergent-based enzymatic method. Low-density lipoprotein cholesterol (LDL-C) was calculated using the Friedewald equation.

Haemoglobin A1c (HbA1c) was measured via capillary electrophoresis (Cap2FP, Sebia, Lisses, France). Glucoregulatory peptides, such as insulin, C-peptide, glucagon, gastric inhibitory polypeptide (GIP), total glucagon-like peptide-1 (GLP-1), and amylin, were quantified using the MILLIPLEX^®^ Human Metabolic Hormone Panel (HMH3-34k, Merck KGaA, Darmstadt, Germany). Assays were run on a Luminex^®^ MAGIPIX^®^ analyser (Luminex Corporation, Austin, TX, USA), and data were analysed using the MILLIPLEX^®^ Analyst 5.1 software (Luminex Corporation, Austin, TX, USA). All analytes’ intra- and inter-assay coefficients of variation were <10% and <15%, respectively, except for amylin (<25%).

Insulin resistance (HOMA2-IR) and β-cell function (HOMA2-B) were calculated using fasting insulin and glucose concentrations via the HOMA Calculator (University of Oxford, Oxford, UK, version 2.2.3). All blood analyses, except Milliplex, were conducted in the Liggins Analytical Lab, University of Auckland. Milliplex assays were conducted at the School of Biological Sciences, University of Auckland.

### 2.3. Sample Analysis

#### 2.3.1. Shotgun Metagenomics

DNA extractions were performed with 180 mg faecal aliquots using the NucleoSpin DNA Stool Kit (Macherey-Nagel, Düren, Germany). Extractions were performed following the manufacturer’s instructions, except bead beating was performed using the Qiagen Tissue Lyser (Qiagen, Hilden, Germany) at 30 Hz for 5 min instead of the recommended instrument. Negative extraction controls were performed using 180 µL of UltraPure distilled DNase/RNase-free water (Thermo Fisher Scientific, Waltham, MA, USA). DNA purity and concentration (ng/µL) were analysed using the Implen NanoPhotometer (Nanodrop Technologies Inc., Wilmington, DE, USA).

DNA extracts were normalised to 5 ng/µL before submission to Auckland Genomics Ltd. for shotgun metagenome sequencing. DNA library construction was carried out using Pureplex Unique Dual Index library prep (Seqwell, Beverly, MA, USA), following the manufacturer’s instructions. The final library was normalised and pooled, and the size was selected using SPRI beads at 0.5× concentration. Three molecular-grade water controls were used to control cross-well contamination during library preparation. The final pooled library was sequenced using an S1 Reagent Kit v1.5 (Illumina, San Diego, CA, USA) (2 × 150 bp paired-end sequencing) on a NovaSeq 6000 (Illumina, San Diego, CA, USA) at Livestock Improvement Corporation (Hamilton, New Zealand).

#### 2.3.2. Untargeted Metabolomics

Untargeted metabolomic analyses of participants’ plasma lipid and polar metabolite profiles were conducted using Q-TOF analysis with two monophasic extraction protocols, as previously published [36]. Briefly, lipids were extracted from 10 µL of pre-thawed plasma (maintained at 4 °C) and mixed with 100 µL of pre-chilled butanol:methanol (1:1, *v*/*v*). The resulting mixture was sonicated for 5 min to improve extraction efficiency, then centrifuged at 11,000 rpm for 10 min at 4 °C. The supernatant was collected into HPLC vials and stored at −80 °C until analysis. For polar metabolites, the extraction procedure was as follows: 50 µL of pre-thawed plasma was mixed with 450 µL of pre-chilled acetonitrile:water (9:1, *v*/*v*). This was followed by sonication for 5 min and centrifugation at 11,000 rpm for 10 min at 4 °C; the supernatant was then transferred to HPLC vials and stored at −80 °C until analysis.

All samples we pre-thawed at 4 °C and analysed using a Shimadzu Nexera X2 UHPLC system coupled to an LCMS-9030 Q-TOF mass spectrometer (Shimadzu Scientific Instruments, Columbia, MD, USA). Lipid profiling and chromatographic separation were performed on an Acquity CSH™ C18 1.7 µm, 2.1 mm × 100 mm column (Waters, Milford, MA, USA), with a mobile phase solvent system composed of solvent A: H_2_O/acetonitrile/isopropanol (5:3:2, *v*/*v*/*v*) with 10 mM ammonium formate, and solvent B: H_2_O/acetonitrile/isopropanol (1:9:90, *v*/*v*/*v*) with 10 mM ammonium formate. Polar metabolite profiling and chromatographic separation were carried out on a Thermo Accucore HILIC 2.6 μm, 2.1 × 100 mm column (Thermo Fisher Scientific, Waltham, MA, USA), using a solvent system composed of solvent A: H_2_O with 10 mM ammonium formate, and solvent B: acetonitrile with 0.1% formic acid. Both analytical procedures implemented a 4 µL injection volume with a 400 µL/min flow rate using previously published HPLC gradient programs [36]. Full MS1 spectra at a resolution of 30,000 were measured over the range 250–1250 *m*/*z* for lipids and 70–1000 *m*/*z* for polar metabolites. Using a “sequential window acquisition of all theoretical fragment-ion spectra” (SWATH-MS) protocol for data-independent acquisition (DIA) of MS2 spectra, 20 *m*/*z* spectra sections, from 300 to 1100 *m*/*z* for lipids and 70 to 900 *m*/*z* for polar metabolites, were subsequently collected to obtain a series of overlapping peak windows, with a cycle time of 0.6 s and a normalised collision energy of 25 units. A source voltage of ±4.0 kV for respective positive and negative electrospray ionisation modes was set, and a nebulising gas flow of 2.0 L/min, heater gas flow of 10 L/min, interface temperature of 300 °C, drying gas flow of 10 L/min, desolvation line temperature of 250 °C, and heater block temperature of 400 °C were used within the methods, utilising nitrogen as the inert gas.

### 2.4. Data Preprocessing

#### 2.4.1. Clinical and Biochemical Characteristic Preprocessing

Raw clinical and biochemical characteristics were preprocessed to ensure comparability across outcomes and compatibility with the multi-block framework. All numeric traits were transformed as follows. Skewness in variables such as waist circumference, liver enzymes (ALT, AST, GGT), and lipid concentrations (TG, HDL-C, LDL-C) was reduced via a log transformation. All raw or log-transformed features were then centred to a zero mean and scaled to unit variance. This generated a normalised dataset for multi-block Partial Least Squares (mb-PLS) and sparse multi-block Partial Least Squares (mb-sPLS) modelling.

#### 2.4.2. Shotgun Metagenomics Sequence Processing

Raw sequencing reads were processed for downstream analysis following a quality control pipeline. Initially, BBDuk from the BBMAP suite (version 39.01-GCC-11.3.0, Joint Genome Institute, Berkeley, CA, USA) [37] was utilised to remove adapter sequences, Phix contaminants, and low-quality base pairs from both ends of each read. Next, human DNA was filtered out using BBTools and a custom human genome reference (hg19_main_mask_ribo_animal_allplant_allfungus.fa.gz) [38]. Quality-controlled, host-filtered paired-end reads were profiled with MetaPhlan MetaPhlan (version 4.1.0, Harvard T.H. Chan School of Public Health, Boston, MA, USA) against the ChocoPhlAn SGB database (mpa_vJan25_CHOCOPhlAnSGB_202503) using the ignoring eukaryotes flag. The resulting relative abundance profiles were merged into a single abundance table. The same cleaned, host-filtered reads were concatenated and processed with HUMAnN (version 3.8, Harvard T.H. Chan School of Public Health, Boston, MA, USA) using the ChocoPhlAn (nucleotide) and UniRef 90 (protein) databases for functional profiling. Downstream steps included normalising results using the humann_renorm_table utility script. For functional categorisation, gene families were regrouped into KEGG (Kyoto Encyclopedia of Genes and Genomes) pathways using the map_ko_uniref90 reference with the human_regroup_table utility script, producing regrouped gene family and pathway abundance files. Both raw and relative abundance profiles were subsequently assembled into phyloseq objects (phyloseq version 1.48.0) [39] in RStudio, RStudio (version 4.4.0, Posit Software, Boston, MA, USA) [40].

#### 2.4.3. Metabolomics Data Processing

Raw data files generated from the Q-TOF platform were converted to the centroid mzML format using the Shimadzu file converter (Shimadzu Scientific Instruments, Kyoto, Japan). Processing utilised the open-access software package MS-DIAL (version 4.9, RIKEN Center for Sustainable Resource Science, Yokohama, Japan) [41]. The software executed peak detection, retention time alignment, grouping, and gap filling, as well as run-order and batch-effect correction via the LOWESS algorithm integrated within MS-DIAL [41]. MS-DIAL searched and annotated the acquired DIA MS/MS spectral data against a built-in lipid library containing 257,000 in silico-generated MS/MS fragmentation spectra and against publicly available (Fiehn HILIC) and in-house (AgResearch) libraries for polar metabolites. Before exporting, QC samples were employed for loess-based adjustments to account for run-order effects. Exported data matrices were manually cleaned of unreliably measured peaks relative to QC samples via the relative standard deviation (RSD > 0.3) and had extraction solvent and mobile phase prominent peaks removed. The final data matrices were used for downstream statistical analysis.

### 2.5. Bioinformatics and Statistical Analysis

#### 2.5.1. Univariate Analysis of the Multi-Omic Dataset

We applied differential abundance analysis using the ANCOM-BC2 (version 2.6.0) framework [42,43] to identify microbial (OTU, KEGG) and metabolomic (polar metabolite, lipid) features that varied with self-reported ethnicity while adjusting for covariates. For each data block, we used raw polar and lipid peak intensities and raw read counts (OTU, KEGG). We fitted a fixed-effects model with ethnicity as the primary predictor and included age, sex, body mass index (BMI), and fasting glycaemic status as covariates. The group was set to ethnicity to detect structural zeros, and a prevalence cutoff of 0.10 was applied to filter out rare features. The Holm (Holm–Bonferroni) procedure was used to control the error rate across features. Model fitting was carried out in two stages: first, the iterative (MLE/REML) algorithm was run with a convergence tolerance of 0.01 for 20 iterations max; second, the expectation-maximisation algorithm was utilised with a tolerance of 1 × 10^−5^ for up to 100 iterations. Results of this analysis are available in Supplementary Figure S1.

#### 2.5.2. Integrated Analysis of Clinical, Metagenomic, and Metabolomic Data Using Mixomics

This multi-omics study used four datasets representing different omics data blocks: (1) polar metabolite data, (2) lipid data, (3) relative abundance gene families generated by HUMAnN and regrouped into KEGG pathways (KEGG), and (4) relative abundance profiles for bacterial taxonomy at the species level (OTU). The polar and lipid metabolite data were derived from blood plasma, while the metagenome-derived OTU and KEGG datasets were generated from faecal samples.

Preprocessing was conducted separately for the chromatography metabolite data (lipids and polar metabolites) and the metagenomic relative abundance-based datasets (OTU and KEGG). Because the lipid and polar metabolite datasets contained missing values, represented as zeros, across 302 lipid features (in 36 of 171 participants) and 28 polar metabolite features (in 37 of 171 participants) due to MS pressure fluctuations with sample injections, zeros were considered missing and imputed using the KNN function from the impute package (version 1.78.0) [44]. Subsequently, the nearZeroVar function was used, followed by log transformation, mean-centring, and scaling, as recommended [45]. Features with proportional counts below 0.001% across all samples were filtered for the OTU and KEGG datasets. Subsequently, the nearZeroVar function from the Caret package (version 7.0.1) was applied to the remaining features [46]. Ungrouped gene families (features not assigned to KEGG families by the HUMAnN utility) were filtered to focus on biologically interpretable features. To account for compositionality, we applied a centred log-ratio (CLR) transformation using the clr function from the microbiome package (version 1.26.0) [47], as recommended [48]. Each data block initially contained the following number of features: 47 polar metabolites; 375 lipids; 118,274 KEGG orthologs; and 1559 microbial OTUs. Following the preprocessing steps, the final dataset featured 47 polar metabolites, 375 lipids, 1284 KEGG orthologs, and 365 microbial OTUs.

We conducted both multi-block partial least squares (MB-PLS) and sparse variance (MB-sPLS) using the mixOmics package (version 6.28.0) [28] to integrate our multi-omics data with the continuous clinical variables (Table 1). MB-PLS performed supervised regression across our multi-omic dataset (polar, lipid, KEGG, and OTUs), using a fully connected design matrix to allow for the modelling of relationships between blocks. Using two components, we employed both the MB-PLS and MB-sPLS models for each clinical variable. In the sparse model, block-specific keepX parameters were applied for variable selection and to reduce dataset dimensionality: 2 features for polar, 20 for lipid, 50 for KEGG, and 20 for the species (OTU) block, representing ~5% of features from each block. Each clinical variable was analysed independently, on scaled and centred variables. Across clinical variables, the MB-sPLS models demonstrated improved stability and performance to varying degrees compared to the standard PLS approach, supporting the decision to utilise the sparse methodology for downstream analysis. To allow for the emergence of other, more informative signals from the dataset, three features (trigonelline, caffeine, and theophylline) were excluded from this analysis, as they proved to be the most discriminant features within the polar metabolites for every clinical variable. To assess the contribution of individual features to each clinical variable, we examined block-specific loadings for component 1 from the MB-sPLS models. We extracted all non-zero loading features for each block and recorded the frequency with which each feature was retained across the 35 clinical variables, which can be viewed in Supplementary Table S1. The percentage variance explained by each omics block across the two components for each clinical parameter can be viewed in Supplementary Figure S2.

To assess for discrimination by sex, we first applied PLS-DA to each omic block (polar, lipid, KEGG, and OTUs) using the mixOmics package. The models were tuned via repeated 5-fold cross-validation to reduce the balanced error rate. However, the balanced and classification error rates were poor across all blocks, except for polar metabolites. Therefore, we focused the subsequent analysis on polar metabolites only. We applied sparse PLS-DA (sPLS-DA) with a grid search for the optimal variable selection (keepX = 5–45, by 5 across four components) to optimise the balanced error rate (BER). The final model was assessed based on the overall error rate, BER, class-specific misclassifications, and receiver operating characteristic (ROC) curves using area under the curve (AUC) analysis.

The multi-omics DIABLO analysis [27], implemented within mixOmics [28], requires a design matrix to define the relationships between all four omics data blocks numerically. We utilised a data-driven approach using partial least squares (PLS) to compute the correlations between blocks, subsequently used for the design matrix. Before model development, the dataset was stratified and randomly partitioned into training (80%) and testing (20%) sets to ensure representative outcome group distributions across both subsets. Initial model performance was assessed using a PLS-DA model with 10 components. The default setting for cross-validation used the M-fold strategy (10 folds), repeated 100 times to determine the optimal number of components and the distance measure for the model. Four components and maximum distance performed the best according to the BER. Feature selection was performed using the supervised block sPLS-DA approach. The keepX parameter was set up using a grid search covering a wide range of values appropriate for each dataset. Subsequently, the keepX parameter was tuned through 10-fold cross-validation and repeated 50 times. The final DIABLO model was built using optimal keepX values, and performance was assessed according to the BER and classification error rates. The predictive ability of the final DIABLO models was tested on the unused test dataset using confusion matrices. This analysis used ethnicity (Asian Chinese vs. European Caucasian) and glycaemic status (normoglycaemia vs. prediabetes) as the outcome variables.

## 3. Results

### 3.1. Multi-Omic Signatures of Ethnicity

The supervised DIABLO model using sparsity selected for 10 of 47 polar metabolites, 250 of 375 lipids, 50 of 1284 KEGG features, and 100 of 365 OTUs on the first component and effectively discriminated between Asian Chinese and European Caucasian participants. The model achieved a balanced error rate (BER) of 0.22 and correctly classified 26 of 34 test samples (16 of 19 Asian Chinese; 10 of 15 European Caucasians). Furthermore, ROC analysis further supported the model’s robust discriminative performance (Supplementary Figure S3) within both the metabolite and microbial blocks (polar AUC = 0.96; lipid = 0.87; KEGG = 0.81; OTU = 0.86).

Block-specific error rates across the four latent components revealed specific patterns. Polar metabolites consistently performed well, with the overall error rate improving across components 1 to 4. Likewise, the lipid block showed a modest reduction in error rate, and the KEGG block was stable. The OTU error rates declined from 0.262 to 0.219, reflecting increased discriminant strength in later components. Class-specific trends reflect this consistent separation. In the polar block, Asian Chinese misclassification rates declined across the components, whereas rates for European Caucasians remained stable. The lipid block demonstrated more variability. However, error rates for Asian Chinese participants were lower than those for European Caucasians. The KEGG and OTU blocks followed similar patterns, with error rates for both groups decreasing by the final component (Supplementary Figure S4).

**Figure 2:**
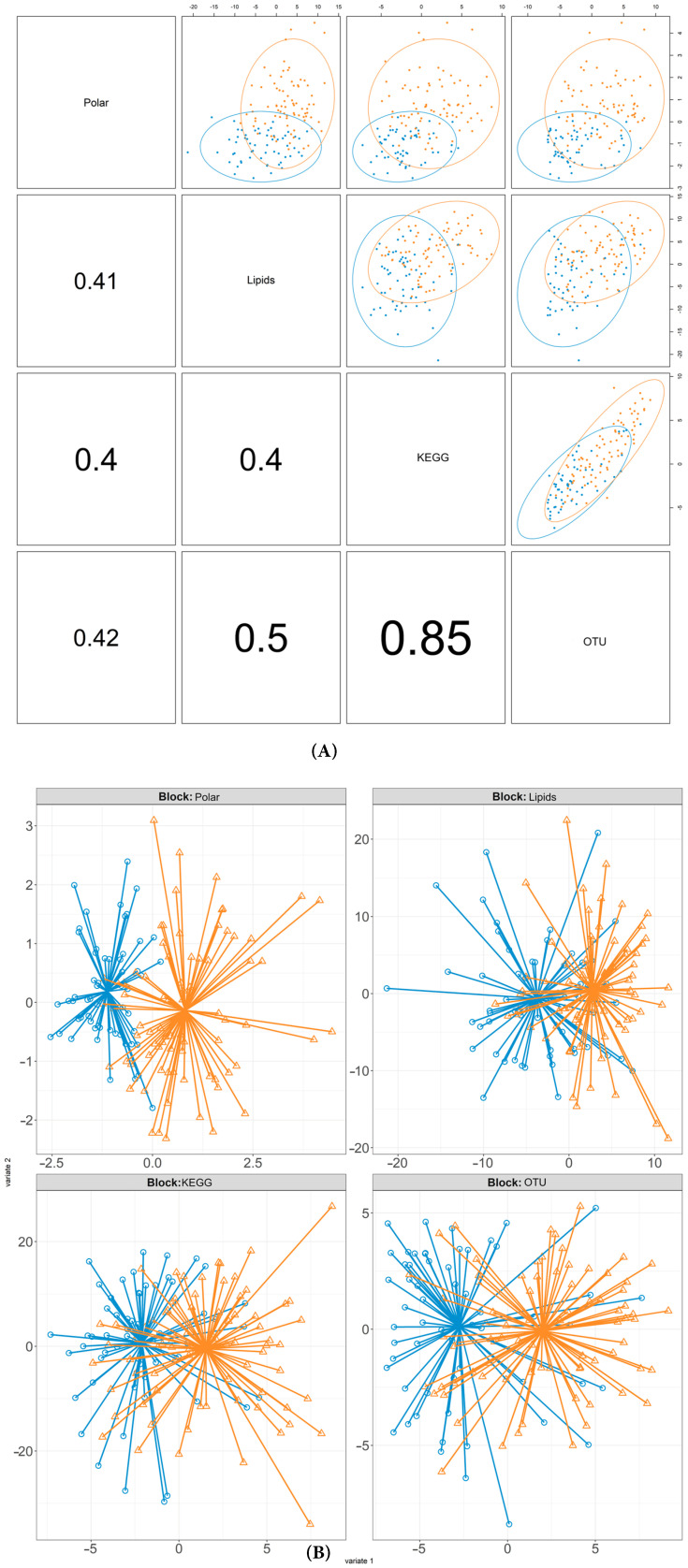
Influence of ethnicity. (**A**) DIABLO diagnostic plots showing multi-omics data integration according to ethnicity, with the most substantial discrimination between Asian Chinese and European Caucasian cohorts based on latent components from all the datasets. The upper right of the figure contains scatter plots, coloured by group types, with ellipses representing 95% confidence. Values to the lower left represent Pearson correlation coefficients between the first components from each dataset. (**B**) Diagnostic plots visualising samples projected on the latent components, showing weak discrimination by each block (data type). Colours distinguish samples from Asian Chinese (orange) and European Caucasian (blue) cohorts.

Consistent with these results, the ethnicity-based DIABLO diagnostic plot analyses further highlighted significant differences between the Asian Chinese and European Caucasian cohorts across multiple datasets (Figure 2). All data blocks, including metagenomic (OTU, KEGG) and metabolomic (lipids, polar) data, demonstrated evidence of discrimination between ethnicities. The pairwise scatterplots further illustrated this trend, with distinct clustering by ethnicity. Figure 2B visualises individual projections across blocks, clearly separating Asian Chinese and European Caucasian participants. This consistent discrimination across datasets highlights robust, ethnicity-specific signatures analysed through our integrative multi-omics profiling.

**Figure 3:**
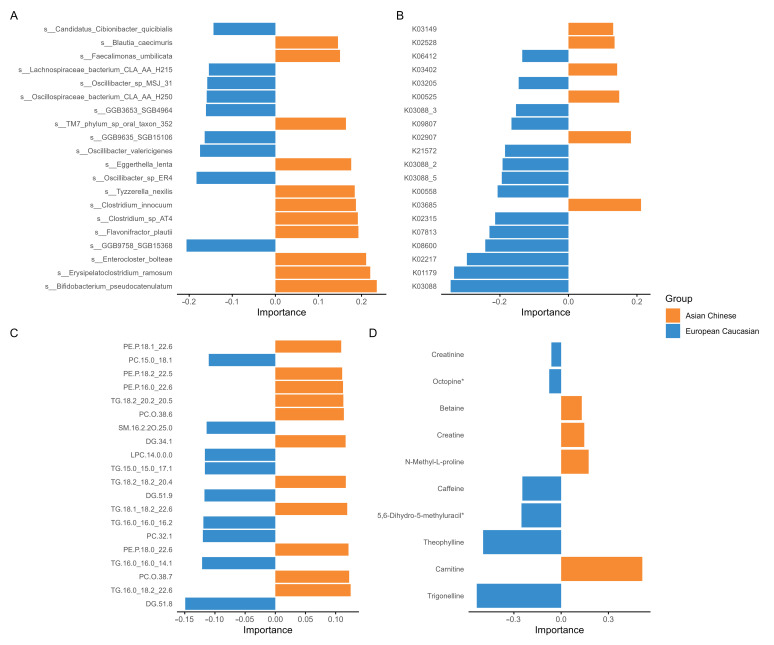
Multivariate analysis of ethnicity using DIABLO. Loading plots represent the top discriminating features for each dataset: (**A**) bacterial species (OTU), (**B**) KEGG pathways, (**C**) lipids, and (**D**) polar metabolites. Features are sorted according to discriminatory strength; bar colour signifies that a feature’s maximal median value is associated with either Asian Chinese (orange) or European Caucasian (blue).

The DIABLO loading analyses revealed ethnicity-associated differences across the metagenomic and metabolomic datasets (Figure 3). Regarding microbial species (OTUs) (Figure 3A), *Bifidobacterium pseudocatenulatum*, *Enterocloster bolteae*, *Erysipelatoclostridium ramosum*, and *Flavonifractor plautii* emerged as the strongest features, with positive importance scores in Asian Chinese participants. By contrast, *Oscillibacter* sp. *ER4*, *GGB9758_SGB15368*, and *Clostridium innocuum* were associated with European Caucasian individuals. Such trends were also present in the functional gene profiles (Figure 3B), where KEGG orthologs, including endoglucanase (K01179), sortase B (K08600), and accessory gene regulator B (K07813), were strongly associated with European Caucasians, and a smaller set of pathways, including RNA polymerase sigma-70 factor, ECF subfamily (K03088), and 26S proteasome regulatory subunit T5 (K03065), characterised Asian Chinese participants.

The DIABLO analyses also uncovered lipidomic signatures differentiating between the European Caucasian and Asian Chinese groups (Figure 3C). Among the Asian Chinese cohort, several lipids demonstrated strong associations. Triglyceride species TG.16:0_18:2_22:6, TG.18:1_18:2_22:6, TG.18:2_18:2_20:4, and TG.18:2_20:2_20:5 and ether-linked phosphatidylethanolamines including PE.P-18:0_22:6 and PE.P-18:2_22:5, as well as phosphatidylcholines such as PC.O-38:7 and PC.O-38:6, were all more abundant and specifically associated with this group. Other notable associations included diacylglycerol DG.34:1 and PE.P.16:0_22:6. The direction and loading values of these associations indicate their relevance to the Asian Chinese group.

Conversely, in the European Caucasian cohort, the dominant associations were noted with lipid species such as DG.51:8, DG.51:9, TG.16:0_16:0_14:1, TG.15:0_15:0_17:1, PC.32:1, PC.15:0_18:1, and LPC.14:0.0.0. Other species, such as SM.16:2;2O/25:0 and TG.16:0_16:0_16:2, were also associated with this group.

Several polar metabolites had evident ethnicity-related associations. For example, trigonelline exhibited the highest loading value and was associated with European Caucasians, while carnitine showed a significant positive association with the Asian Chinese group. Other metabolites, such as theophylline, octopine, creatinine, and caffeine, were positively associated with European Caucasians, whereas betaine and N-methyl-L-proline showed positive associations with the Asian Chinese group (Figure 3D). All features displayed in the loading plots are listed in Supplementary Tables S2–S5.

### 3.2. Cross-Omic Microbial–Metabolite Hubs in Ethnicity-Stratified Analysis

To identify cross-omics features underpinning ethnicity, we focused on correlations derived from the DIABLO model discriminating between Asian Chinese and European Caucasians, specifically, amongst features selected as discriminatory by the ethnicity model. The glycaemia-stratified model did not generate robust results, and thus, the following results are restricted to ethnicity-associated correlations between microbial, functional, and metabolic processes.

**Figure 4:**
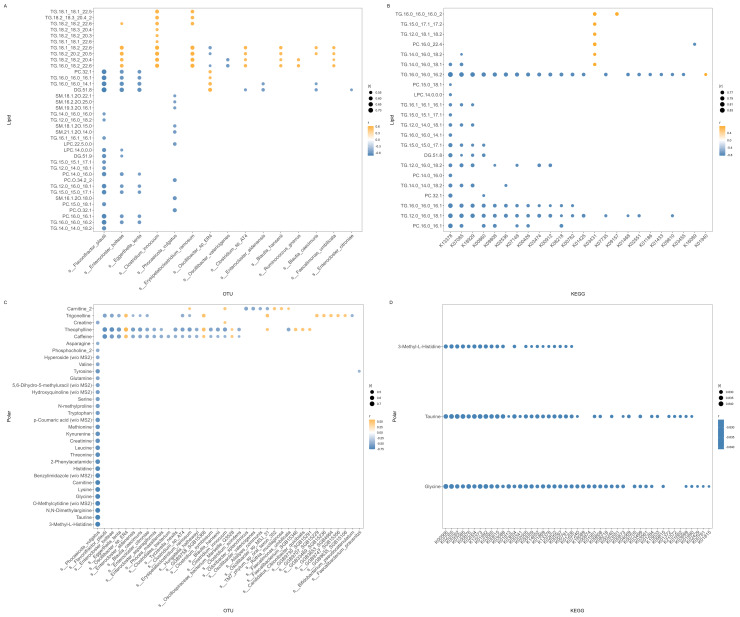
Top cross-omic correlations from the ethnicity DIABLO model. The correlation matrix was derived from the supervised DIABLO model discriminating Asian Chinese vs. European Caucasian participants along components 1–4 and consists of four block pairs: Lipid–OTU (**A**), Lipid–KEGG (**B**), Polar–OUT (**C**), and Polar–KEGG (**D**). The top 100 feature pairs with the largest correlation are displayed as dots, sized by correlation value magnitude and coloured from blue to orange for negative-to-positive values. Full feature details and correlation values are in Supplementary Tables S6–S9.

Across all four cross-omics pairings, we identified distinct and consistent cross-block correlations (Figure 4). In each block pairing, metabolic and microbial features consistently emerged as correlation hubs (Supplementary Figure S5).

Lipid–OTU correlations (Figure 4A) ranged from –0.725 to 0.619. *Flavinofractor plautti* emerged as a key hub, negatively associated with DG.51.8, PC.32.1, TG_16:0_16:0_14.1, TG.16:0_16:0_16.2, and TG.16:0_16:0_16.1. Conversely, *Clostridium innocuum* and *Erysipelatoclostridium ramosum* emerged as key hubs that were positively associated with LCFA and very-long-chain fatty acid (VLCFA)-containing TGs (e.g., TG.18:1_18:2_22:6, TG.16:0_18:2_22:6, TG.18:1_18:2_22:6, TG.18:2_20:2_20.50). Lipid classes such as TGs and PCs containing both LCFA and VLCFAs emerged as notable hubs on the lipid side, and with *F. plautti*, *C. innocuum*, *Enterocloster bolteae*, and *Eggerthella lenta* as key microbial hubs on the OTU side. Interestingly, *E. bolteae* exhibited bidirectional associations, negatively correlating with DG.51.8 and TGs TG.16.0_16.0_14.1 and TG 16.0_16.0_16.1 while also positively correlating with TG.18.2_20.2_20.5.

The lipid–KEGG correlations (Figure 4B) ranged from –0.833 to 0.769. NADH-quinone oxidoreductase subunit C/D (K13378) emerged as a negative hub, correlating with several triglycerides including TG.16.0_16.0_16.2, TG.16.0_16.0_16.1, TG.12.0_16.0_18.1, TG.15.0_15.0_17.1, TG.14.0_14.0_18.2, and TG.12.0_16.0_18.2, as well as diglyceride DG.51.8 and phosphatidylcholines PC.32.1, PC.16.0_16.1, and PC.14.0_16.0.

Conversely, among the primarily negative correlations involving the KEGG and lipid blocks, phosphoglucosamine mutase (K03431) generated the strongest positive links to several triglycerides, including TG.16.0_16.0_16.0_2, TG.15.0_17.1_17.2, TG.12.0_18.1_18.2, TG.14.0_16.0_18.1, and TG.14.0_16.0_18.2, as well as with PC.16.0_22.4, and arginosuccinate synthase (K01940) was positively correlated with TG.16.0_16.0_16.2. Long-chain fatty acid (LCFA) triglycerides (TGs) and phosphatidylcholines (PCs) were the most prominent lipids. In contrast, redox enzymes and kinases emerged as key KEGG hubs.

As for the polar–OTU-blocks, correlations ranged from –0.7563 to 0.5885 (Figure 4D). The strongest negative associations, all of which were anchored on *Phocaeicola vulgatus*, negatively correlated with a wide range of amino acid derivatives, 3-methyl-histidine, taurine, N-dimethylarginine, o-methylcytidine, and glycine. This is in line with the trends observed for the KEGG–polar block. Conversely, the top five positive correlations included coffee intake-associated metabolites, for example, *Oscillibacter_sp_ER4s* correlations with caffeine and trigonelline, and *GGB9758_SGB15368* and trigonelline. Amino acids formed negatively correlated hubs, whereas xanthine derivatives (e.g., theophylline, caffeine) and trigonelline emerged as positive hubs, with OTUs *P. vulgatus* and *Oscillibacter* acting as negative and positive hubs. Notably, carnitine emerged with a species-specific correlation, negatively correlated with *Oscillibacter* and positively correlated with *TM7_phylum_sp_oral_taxon_352*. Unlike the trends observed in the OTU–lipid correlation, the OTU–polar metabolite correlations highlighted amino acid depletion and xanthinin and alkaloid enrichment.

Finally, the polar–KEGG correlations (Figure 4D) ranged from –0.8411 to –0.8262, forming largely uniform negative associations, without the positive outliers seen in the KEGG–lipid block. The strongest pairs emerged as tRNA-uridine 2-sulfurtransferase (K00566) and glycine, elongation factor G (K02355) and glycine, glutamate dehydrogenase (K00262) and taurine, elongation factor G (K02355) and taurine, as well as a putative transport protein (K07085) and taurine. tRNA-modifying enzymes, ABC transporters, and dehydrogenases emerged as recurring hubs in the KEGG block, uniformly negatively correlated with amino acid metabolites (glycine, taurine, and 3-methyl-L-histidine). The largely negative correlations here mirrored the trends observed in the KEGG–lipid correlation block. Notably, all KEGG orthologs in the correlation pairs were annotated with *Phocaeicola vulgatus,* heavily implicating this bacterium’s metabolism in these associations. All features and correlation values are available in Supplementary Tables S6–S9.

### 3.3. Influence of Glycaemic Status on Multi-Omics Datasets

We applied the DIABLO framework to classify fasting glycaemic status (normoglycaemia vs. prediabetes). However, the model failed to achieve meaningful separation and proved unstable. Global error rates using the maximum distance hovered around 24–25% across the four omics blocks, and the overall error rate remained at 0.50 under the majority, weighted, and average voting schemes. Class-specific performance was highly skewed: participants with normoglycaemia were effectively always correctly classified (error ≈ 0–0.014 across blocks and components), whereas participants with prediabetes were globally misclassified (error ≈ 0.96–1). Furthermore, feature selection stability was underwhelming, alongside poor AUCs. Thus, we decided not to include the glycaemic status DIABLO model in this manuscript. However, the results and performance metrics of the analysis can be seen in Supplementary Figures S6–S9.

### 3.4. Assessing Block-Specific Discrimination: Polar Metabolite-Driven Sex Separation

To assess sex-based discrimination across omics layers, we first applied PLS-DA models to each data block (polar, lipids, KEGG, and OTU), using sex as the outcome. We then applied sparse PLS-DA models separately to each block (polar, lipids, KEGG, and OTUs) to improve feature selection and model stability. Across both the PLS-DA and sPLS-DA models, polar metabolites consistently outperformed the other blocks, with the highest explained variance on component 1 and the lowest error rate across all distance matrices. Lipids followed, explaining less variance and higher error rates, with notably both KEGG and OTU features explaining less variance and noisier classification results. Class-specific error rates demonstrated higher male classification errors compared with females across all blocks. The diagnostic results from these PLS-DA models are available in Supplementary Figure S10. As for the sPLS-DA result, these trends were consistent (Supplementary Figure S11): polar metabolites demonstrated the lowest class-specific error rates, with male error rates dropping to 17.9% and female error rates to 10.6% at the 5th component. Other blocks, such as lipids and OTUs, performed worse, with male error rates of 43% and 49%, respectively. Given these consistent performance results, we focused subsequent analysis on the polar metabolite dataset, which provided the greatest discriminative power, model stability, and interpretability for sex-based separation.

**Figure 5:**
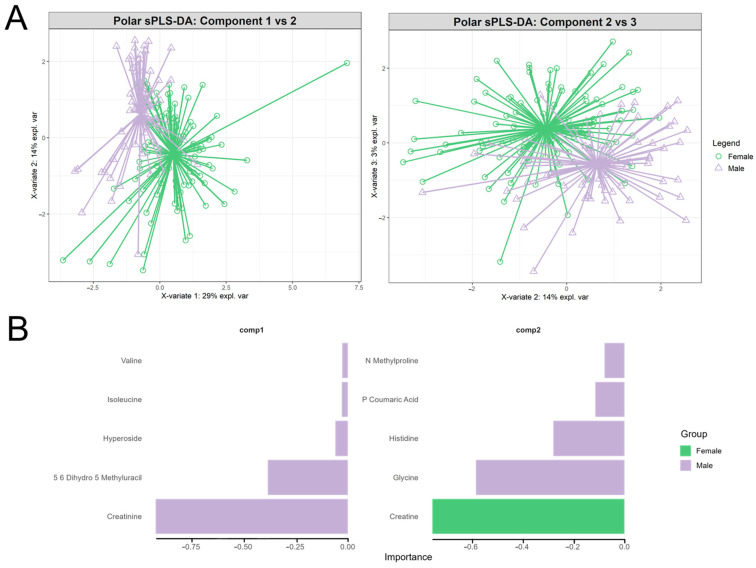
Discrimination by sex using sparse partial least squares discriminant analysis (sPLS-DA) on polar metabolites. (**A**) Sample plots showing the separation of females (green) and males (purple), across components 1 vs. 2 and 2 vs. 3, based on latent variables derived from the polar metabolite dataset. (**B**) Loading plots highlight the top discriminatory polar metabolites contributing to each component, with colour indicating the sex with the higher median abundance.

The sparse PLS-DA results for polar metabolites revealed strong discrimination between sexes, as visualised in the component score plots and variable loadings (Figure 5). Samples formed distinct sex-based clusters, with centroids primarily separated along the axis of component 1. This distinct visual separation reflects component 1’s high explained variance (29%) and strong association with the sex outcome. While component 1 was the primary driver of separation, the distribution of samples along component 2 did capture some additional sources of variance (14%) within or between groups but did not contribute to further separating sexes.

As for features contributing to this separation along component 1, all features were associated with the male group. Key polar metabolites included creatinine, dihydro-5-methyluracil, hyperoside, isoleucine, and valine. Component 2 captured secondary variance, with features in this component associated with female and male groups (Figure 5). The sole feature contributing to the female group and component 2 was creatine, with glycine, histidine, coumaric acid, and N-methyl proline as top contributors to the male group within component 2. The loading plot includes all variables selected by the sPLS-DA model, representing 10 out of the 47 original features.

## 4. Discussion

Here, we build upon the baseline TOFI_Asia study [25,26] with a cross-sectional analysis of a subset of returning participants at the 4-year follow-up point. Specifically, we utilised a multi-omics approach to analyse faecal and plasma samples from 171 Asian Chinese or European Caucasian adults resident in New Zealand, aiming to uncover differences based on ethnicity and/or glycaemic status. In concert with targeted lipidomic and polar metabolite profiling of participant plasma samples, shotgun metagenomic sequencing of faecal samples was employed to determine bacterial community composition and functional potential. Integrating microbiome, lipid, and polar metabolite datasets into a single framework allowed us to recognise interactions between data blocks that may have gone unnoticed if each dataset was analysed in isolation. This multi-omic approach provides a more comprehensive view of how the gut microbial composition may be associated with host metabolic pathways and their joint influence on T2D risk across different ethnic groups, as our findings underscore the importance of integrating ethnicity-based metabolic phenotypes when considering the differential risks for T2D amongst different populations.

### 4.1. Metagenomic Distinctions Are Primarily Driven by Ethnicity and Not Glycaemic Status

Our findings reaffirm the ethnic differences identified in our earlier 16S rRNA gene amplicon study of Asian and Caucasian populations resident in New Zealand [23] and provide extra insight via examination of the functional potential of the gut microbiome. Our previous 16S-based study identified differentially abundant microbial taxa between ethnic groups, with several of these re-identified as discriminatory between ethnicities in the current shotgun metagenomic analysis. Bacterial genera such as *Blautia* and *Roseburia* were differentially abundant in both the earlier 16S study [23] and this metagenomic dataset; for example, *Roseburia* sp. *AF02_12* and *Blautia SGB101324* were identified at the species level in the metagenomics data, and their respective genera were also differentially abundant in the lower-resolution 16S rRNA gene dataset. However, the functional implications of these differences were not explored in the 16S dataset. Our current shotgun metagenomics approach revealed significant associations between functional pathways that may relate to gut microbiome-derived metabolic differences between ethnic groups. The differential abundance analysis (Supplementary Figure S1) supports the results of the DIABLO ethnicity model, where Asian Chinese participants had higher abundances of *E. ramosum*, associated with metabolic dysregulation, and lipid absorption via serotonin pathways [49].

On the functional level (Supplementary Figure S1), we observed an enrichment of several KEGG orthologues, such as K02881 and K03433, in European Caucasians, annotated with the archaeon *Methanobrevibacter smithii*, a key species associated with energy harvest and syntrophic interactions with SCFA-producing bacteria. Recent evidence suggests that the archaeome composition is shaped by geography and lifestyle, with rural residents consuming traditional diets harbouring higher abundances of *M. smithii* [50]. This species plays a central role in the gut microbiome, supporting cross-feeding with SCFA producers such as *Roseburia* and *Prevotella*. Notably, studies have associated the absence of *M. smithii* as a vital factor in severe acute malnutrition, underscoring its role in energy extraction and overall gut health [51]. The decreased abundance of *M. smithii*-associated functions in our Asian Chinese cohort, alongside the reduced abundance of SCFA-producing bacteria (Supplementary Figure S1) such as *Roseburia* [52], *Blautia* [53], and *Faecalimonas umbilicata* [54], may reflect the effects of urbanisation and Westernised lifestyles.

The multi-omics framework also added value by highlighting cross-omics correlations, which cannot be detected through a single-omics approach. This systems-level approach helped to elucidate interactions across multiple datasets, providing a broader overview of how ethnicity or glycaemic status may collectively influence host–microbiome–metabolic phenotypes. Overall, in our multi-omics analysis, shotgun metagenomics offered robust evidence for ethnicity variations, in line with the previous literature [22,55,56,57]—but not glycaemic status, despite prior metagenomic-based findings on glycaemic status [58,59,60].

Our findings uncover distinct microbial signatures that distinguish Asian Chinese from European Caucasian participants, highlighting the importance of bacterial taxa in human health and disease. Furthermore, long-term habitual dietary patterns may drive these cohort-specific differences. Notably, *Bifidobacterium pseudocatenulatum* was positively associated with Asian Chinese participants. This species may improve gut barrier integrity and exert anti-inflammatory action, attributable primarily to the fermentation of plant-derived non-digestible carbohydrates into SCFAs such as acetate [61]. Furthermore, *B. pseudocatenulatum* can impact host metabolic homeostasis, with animal studies showing that specific strains can reduce body fat and improve glycaemic control and insulin sensitivity [62]. As dietary patterns vary across ethnicities, enrichment of *B. pseudocatenulatum* among Asian Chinese participants could reflect diets richer in fibre and resistant starches. Conversely, species with pathogenic potential, including *Erysipelatoclostridium ramosum* and *Enterocloster bolteae*, were also associated with Asian Chinese participants. *E. ramosum* was also identified as more abundant in Asian Chinese participants via the fold-change analysis (Supplementary Figure S1), and it was selected within the multi-block sPLS regression (Supplementary Table S1). *E. ramosum* is an opportunistic pathogen involved in clinical infections and bacteraemia and may promote obesity through Toll-like receptor (TLR)-4 signalling-mediated inflammatory pathways [63]. Furthermore, *E. bolteae* has been associated with chronic liver disease and autism spectrum disorder via the production of ethanol and other neurotoxic metabolites [64,65]. Conversely, we observed elevated abundances of *Oscillibacter* spp. in European Caucasians, and these taxa are considered beneficial, being positively associated with lean body composition, improved metabolic profiles, and anti-inflammatory properties due to SCFA (butyrate) production [66,67]. The higher abundance of *Oscillibacter* sp. among European Caucasians may be due to dietary, lifestyle, or genetic factors affecting the abundance of SCFA producers, impacting host metabolic resilience.

The microbial gene families identified in this analysis and regrouped into KEGG pathways highlight pathways and mechanisms associated with ethnicity differences. In the European Caucasian cohort, genes encoding for proteins such as RNA polymerase sigma-70 factor (K03088) suggest differences in bacterial transcriptional regulation, impacting microbial responses to the host environment, diet, and other stressors. Sigma-70 factors are transcriptional regulators that modulate gene expression in response to environmental stressors such as antimicrobial pressure and host immune responses [68]. K03088 also emerged as a consistently selected KEGG feature across the multi-block sPLS models, reflecting its relevance to differences in transcriptional regulation across groups. Elevated sigma-70 factor activity could reflect a more flexible microbiome resilient to perturbations and impacts of disease vulnerability between ethnic groups. The apparent enrichment of endoglucanase enzymes (K01179) in European Caucasian participants is also of note, as endoglucanases degrade cellulose, facilitating the production of fermentable substrates [69]. Endoglucanase abundance possibly reflects increased plant-derived polysaccharide dietary patterns in European Caucasians compared to the Asian Chinese cohort or an inherent difference in microbial carbohydrate metabolism capacity. K01179 was a discriminant feature consistently selected within the multi-block sPLS regression, reaffirming its relevance in host metabolic functions.

The prevalence of microbial ferritin (K02217) in the European Caucasian cohort is also intriguing, where microbial ferritin production inherently differs between ethnicities and may impact ethnicity-specific health outcomes related to cognitive function and metabolic syndrome. Dysregulation may be associated with cognitive decline, inflammatory conditions, and impaired butyrate production [70,71,72], or it may reflect differences in dietary iron intake. Once again, K02217 was also frequently selected in the sPLS regression, highlighting its associations with ethnicity and host metabolic health parameters. Conversely, the Asian Chinese microbial functional profile was enriched with ribonuclease III (K03685), implicating bacterial RNA processing techniques as ethnicity-dependent, which may impact metabolic flexibility and adaptability within host immune and dietary pressures [73]. RNA processing is integral for bacterial survival, virulence, stress resistance, biofilm formation, and motility; RNase III activity impacts *Salmonella enterica*’s competitive behaviour during infection [73,74], hinting at ethnic differences underpinning bacterial responses to host processes and environmental stressors.

### 4.2. Cross-Omic Correlation Structure Reveals Metabolic and Microbial Hubs

The cross-omic correlation analysis revealed a pattern of consistent negative associations between KEGG orthologs and host lipid and polar metabolites, with only a handful of positive correlations. KEGG feature K13378 (NADH-quinone oxidoreductase subunit C/D) was the most recurring, with strong negative correlations with triglyceride and phosphatidylcholine species. K13378 encodes a key subunit of respiratory complex I, an integral component of electron transfer and ATP synthesis in bacterial and mitochondrial systems [75,76]. The repeated negative associations with host plasma lipid species may reflect a microbial–host metabolic trade-off associated with redox balance and energy metabolism. Furthermore, KEGG features K18929 (L-lactate dehydrogenase), K0860 (adenyl sulphate kinase), and K09808 (lipoprotein-releasing permease) demonstrated a broad range of negative correlations with several lipid classes. Adenyl sulphate kinase is involved in sulphur amino acid metabolism and is linked to cardiovascular risk due to its influence on phosphatidylcholine (PC) biosynthesis [77] and was negatively associated with a single PC species and several triglyceride species.

Notably, multiple triglyceride species, such as TG.18.1_18.2_22.6 and TG.16.0_18.2_22.6, demonstrated strong positive correlations with members of the *‘Clostridium’* genus complex, specifically, *C. innocuum, E. ramosum*, and *E. bolteae*. This is notable due to *E. ramosum*’s documented impact on host metabolism via enhancing intestinal lipid absorption via serotonin-mediated enterochromaffin cell development [49] and promoting high-fat-diet-induced obesity in mouse models [78]. Upregulation of intestinal fatty acid transporters may mechanistically explain the positive associations with host triglyceride levels. Furthermore, *E. bolteae* has been associated with dysbiosis. In a multi-omic study of overweight pregnant women, its abundance was positively associated with a cluster of pro-atherogenic plasma metabolites, such as low-density lipoproteins and triglyceride-associated metabolites [79]. This is in keeping with our observation of positive correlations between *E. bolteae* and long-chain triglyceride species and negative correlations with specific diacylglycerols. *E. bolteae* has also been identified as an ethanol-producing gut bacterium in liver disease and thus contributes to lipid-rich metabolic profiles [64]. The observed negative associations between *E. bolteae* and coffee-associated metabolites (e.g., caffeine, theophylline) imply that coffee drinkers may have lower *E. bolteae* abundances, as coffee intake can be associated with an increased abundance of beneficial taxa (e.g., *Alistipes*, *Faecalibacterium*) and overall diversity, possibly suppressing opportunistic pathogens such as *E. bolteae* [80]. Therefore, *E. bolteae* positive correlations with TGs and negative correlations with coffee-associated metabolites may indicate a less healthy gut environment. The convergence of these species with a cluster of triglycerides, considering triglycerides’ well-known association with cardiovascular and diabetes risk [81,82,83], raises the possibility of genus-level metabolic manipulation influencing host lipid dynamics. Indeed, phosphoglucosamine mutase is a bacterial enzyme in the peptidoglycan precursor biosynthesis pathway, which was positively associated with specific host lipids in our multi-omic analysis. This likely reflects an increase in Gram-positive microbial communities, which often display high cell wall synthesis activity and an increase in abundance with high-fat diets [78]. Enrichment of this enzyme may reflect an increased abundance of Gram-positive bacteria in a lipid-rich environment [78].

We uncovered a negative correlation between *Flavonifractor pluattii* abundance and host triglyceride levels, consistent with prior research that higher *F. plautii* abundance is associated with lower BMI and triglyceride concentrations and that *F. plautii* is less abundant in the faecal microbiota of obese individuals [84,85,86]. Mechanistically, this is likely attributable to one of its metabolic products, phytosphingosine, activating hepatic metabolism, promoting fatty acid oxidation, and improving lipid metabolism [87].

In our cross-omic analysis, the majority of KEGG orthologs, primarily annotated to *Bacteroides vulgatus*, now *Phocaeicola vulgatus*, were negatively correlated with host polar metabolites. This pattern is consistent in the OTU–polar metabolite correlations, where roughly half of the strongest negative correlations are anchored by *P. vulgatus*, and in line with its role in bile and amino acids, including branched-chain amino acid (BCAA) metabolism [88,89]. *P. vulgatus* was negatively associated with several host-derived polar metabolites such as glycine, taurine, 3-methyl-L-histidine, and BCAAs (valine, leucine, threonine), alongside KEGG orthologs such as glutamate dehydrogenase (K00262) and NADH-quinone oxidoreductase subunits (K13378). This likely stems from *P. vulgatus* bile salt hydrolase activity, which deconjugates amino acids such as glycine and taurine from bile acids [90]. The release of glycine/taurine is subsequently metabolised by gut bacteria such as *Bilophila wadsworthia* and *Alistipes*, which require taurine for growth [90]. The metabolic activity of *P. vulgatus* is expected to decrease the levels of amino acids in the intestines available for host uptake. Furthermore, the gut microbiome produces branched-chain fatty acids from dietary valine, leucine, and isoleucine, which explains the negative association mechanistically [91]. Moreover, we also observed a negative association between *P. vulgatus* and 3-methylhistidine, suggesting microbiome-mediated metabolism of methylated purines.

In summary, *P. vulgatus* levels may reflect a gut microbiome actively recycling bile acids, scavenging nitrogen, and thus decreasing free amino acid and nucleoside levels in its host. Some *P. vulgatus* isolates protect against hyperlipidaemia and colitis [92], while other strains may aggravate cardiac fibrosis and bone loss [93,94]. Interestingly, *P. vulgatus* has also been associated with improved metabolic profiles, where supplementation of mice with *P. vulgatus* was protective against diet-induced obesity [95]. Altogether, our findings cannot easily determine whether *P. vulgatus* is beneficial or deleterious, highlighting the need for strain-resolved functional annotations to understand further its role within host metabolism and the gut microbiome.

### 4.3. Metabolomic Datasets Reinforce Ethnicity over Metabolic Status Separation

Consistent with our previous findings from the baseline TOFI_Asia cohort [25], multi-omic analysis of lipids and polar metabolites obtained from blood plasma successfully differentiated between the European Caucasian and Asian Chinese cohorts (Figure 3 and Figure 4). These results likely reflect ethnicity-linked variations driven by diet, genetics, and gut microbial metabolism influencing host metabolic processes.

Plasma lipidomic profiles highlighted metabolic features associated with ethnicity, reflecting differences in fatty acid metabolism and dietary intake between Asian Chinese and European Caucasian cohorts. The Asian Chinese cohort demonstrates enrichment of triglycerides containing very-long-chain polyunsaturated fatty acids (PUFAs), specifically, TG.16.0_18.2_22.6, TG.18.1_18.2_22.6, TG.18.2_18.2_20.4, and TG.18.2_20.2_20.5, where the 22:6 (docosahexaenoic acid) and 20:4 (arachidonic acid) notations hint at the incorporation of omega-3 and omega-6 fatty acids originating from meat, poultry, eggs, fish, and dairy products [96,97]. Omega-3 and omega-6 fatty acids are biologically active, modulating gene expression and regulating cell membranes [98]. PUFAs are of major importance in human health, specifically, in the prevention of neurological disorders in adults, and in the aetiology of metabolic diseases such as diabesity and obesity [98]. The co-occurring elevation of ether-linked phospholipids PE.P.18.0_22.6, PE.P.18.2_22.5, PE.P.16.0_22.6, PC.O.38.7, and PC.O.38.6 may hint at enhanced plasmalogen biosynthesis through peroxisomal alkyl-dihydroxyacetone phosphate synthase activity [99,100]. Ether-lipids play a key role in maintaining cell membrane fluidity, cellular signalling, and other cellular processes [101].

In contrast, the European Caucasian cohort was predominantly enriched with saturated and monounsaturated lipid species such as TG.16.0_16.0_14.1, TG.15.0_15.0_17.1, TG.16.0_16.0_16.2, and PC.15.0_18., in line with Western dietary patterns characterised by high levels of saturated fat consumption from dairy and meat products [102,103]. Furthermore, the elevated levels of lysophosphatidylcholine.14.0.0.0, which plays a key role in the development of atherosclerosis and inflammatory diseases [104], may reflect the pro-inflammatory states associated with Western diets [105].

Polar metabolites, notably, trigonelline and carnitine, revealed differential metabolic profiles based on ethnicity. Trigonelline, theophylline, and caffeine are enriched in European Caucasians and may be biomarkers of coffee intake and muscle metabolism. Furthermore, trigonelline is negatively associated with diabetes and cardiovascular risk through microbiome-mediated pathways [106,107]. A recent study demonstrated that trigonelline can directly inhibit gut bacterial metabolism of choline to TMA, thus preventing TMAO formation [107]. The inhibitory effect on bacterial TMA production may be a mechanistic link for trigonelline’s protective cardiovascular effect, in contrast with the carnitines’ harmful pathway [107].

Conversely, carnitine was elevated in the Asian Chinese cohort, which may underscore ethnicity-specific dietary or microbial metabolism patterns. The conversion of carnitine into TMA by the gut microbiome, which is consequently metabolised into the pro-atherogenic trimethylamine-N oxide (TMAO) in the host liver, aligns with observed elevated stroke risks in Asian populations [108,109,110,111]. Moreover, betaine was also elevated in the Asian Chinese cohort and, like carnitine, can be metabolised into TMA and subsequently into TMAO [111,112]. Notably, Qin et al. [113] identified 3-hydroxybutyrylcarnitine as elevated in people with T2D. As such, this metabolite may serve as a biomarker signifying mitochondrial dysfunction and impaired fatty acid β-oxidation, both features integral to T2D pathology [113]. Carnitine was repeatedly selected within the multi-block sPLS regression models, present in 29 of the 35 outcome variables, reinforcing its centrality in ethnicity and clinical health parameters, including those associated with adiposity and insulin resistance, as seen in Supplementary Table S1.

### 4.4. Sex-Associated Variation in Polar Metabolite Signatures

The sPLS-DA models revealed that polar metabolites consistently and more effectively discriminated between male and female participants, with component 1 accounting for 29% of the variance. Features driving the sex-associated separation were primarily male-associated, including creatinine, isoleucine, valine, and 5-methyluracil. These findings align with sex-based biochemical differences, where serum creatinine levels are typically elevated in males due to greater muscle mass and renal handling [114]. Branched-chain amino acids (isoleucine, valine) were also elevated in males, a trend also observed in a population-level study, which observed higher circulating BCAA levels in Chinese males despite minimal impact from dietary intake [115]. The only feature correlated with females was creatine; lower creatine reserves are reported in females compared to males [116].

### 4.5. Methodological Considerations

This study compared two ethnic groups (Asian Chinese and European Caucasian) and two glycaemic states (normoglycaemia and prediabetes). These comparisons allowed for insights into specific metabolic differences, though the extent to which our findings can be generalised to other populations and individuals across the range of glycaemic dysregulation (e.g., frank T2D) is somewhat limited. The class imbalance within the glycaemic state group is likely responsible for the poor discriminative performance within our glycaemic status-based DIABLO model, likely obfuscating biological signals. Furthermore, using faecal samples for metagenomics but blood plasma for metabolomics leads to differing time scales, as plasma levels can shift rapidly following changes in diet or metabolism. In contrast, the gut microbiome may shift over days or weeks. Likewise, by assessing the bloodstream and gut compartments separately, we generated a complementary but separate view of host–microbe interactions.

As this study comprised a cross-sectional analysis, we cannot assume causal or temporal relationships, with longitudinal data necessary to further our understanding of how these relationships operate over time. Despite this, a key strength of our multi-omics framework is that it integrates multiple data sets in a single analytical model, providing a more comprehensive assessment of host–microbe interactions than would be possible with separate, single-omics approaches. This system-level perspective enhances our ability to identify potential biomarkers and mechanistic pathways relevant to metabolic health in diverse populations. Furthermore, a significant strength of this study was the use of a multi-block, sparse PLS methodology, which enables the detection of biological signals across the utilised omic layers. By applying this methodology, we offer a broader overview of gut–host metabolic relationships and their relevance within an ethnically diverse cohort.

## 5. Conclusions

This multi-omic analysis demonstrates that ethnicity is a primary driver of metabolic and microbial variation between the Asian Chinese and European Caucasian cohorts. Polar metabolites demonstrated the highest discriminatory power (AUC = 0.96), followed by lipids (AUC = 0.87), with microbial features contributing somewhat less robustly (KEGG AUC = 0.81; OTU AUC = 0.86). The ethnicity-based DIABLO model achieved 76.5% classification accuracy, identifying ethnicity-specific metabolic signals: trigonelline characterised European Caucasians, whereas carnitine characterised Asian Chinese participants. These metabolites are directly linked to differential disease risk; trigonelline inversely correlates with diabetes risk, while carnitine is microbially converted to pro-atherogenic TMAO.

Cross-omic correlations revealed mechanistic insight into microbe–metabolite interactions. *Phocaeicola vulgatus* exhibited strong negative correlations with glycine, taurine, and branched-chain amino acids, in line with its bile salt hydrolase activity, deconjugating amino acids from bile acids. *Erysipelatoclostridium ramosum* and *Clostridium innocuum* were positively correlated with polyunsaturated triglycerides, supporting *E. ramosum*’s role in enhancing intestinal lipid absorption. *Flavonifactor plautti* was negatively correlated with saturated triglycerides, aligning with its production of phytosphingosine, which promotes hepatic fatty acid oxidation.

The ethnicity-specific microbe–metabolite profiles provide mechanistic insight into differential T2D susceptibility between Asian Chinese and European Caucasian populations. The integration of our multi-omic dataset revealed metabolic networks where microbial metabolism may directly modulate host lipid and amino acid profiles, establishing biomarkers for future population-specific metabolic disease risk assessment.

## Figures and Tables

**Table 1:** Clinical and biochemical characteristics of the entire cohort (*n* = 171) and ethnicity and glycaemic state subgroups 2.

Variable	Asian Chinese (*n* = 99)	European Caucasian (*n* = 72)	*p*-Value	Normo-Glycaemia (*n* = 130)	Prediabetes (*n* = 41)	*p*-Value
M:F ratio	40:59	30:42	**0.99**	43:87	27:14	**<0.01**
Age (y)	46.6 ± 12.8	51.2 ± 15.6	**0.05**	46.9 ± 14.2	53.8 ± 13.1	**<0.01**
Body weight (kg)	73.2 ± 13	81.3 ± 16.7	**<0.01**	75.2 ± 15.6	81.1 ± 12.8	**<0.01**
Height (m)	1.7 ± 0.1	1.7 ± 0.1	**<0.01**	1.7 ± 0.1	1.7 ± 0.1	0.27
BMI (kg m^−2^)	26.7 ± 3.7	27.1 ± 4.7	0.77	26.5 ± 4.4	28.1 ± 3.3	**0.01**
Waist circumf. (cm)	89.3 ± 10.4	93.2 ± 13.7	0.09	89.1 ± 11.8	96.8 ± 11.1	**<0.01**
Hip circumf. (cm)	102.1 ± 6.6	107.6 ± 10	**<0.01**	104.1 ± 8.9	105.3 ± 7.6	0.42
SBP (mm Hg)	123 ± 17.3	117.5 ± 16.7	**0.02**	119.1 ± 17.3	125.7 ± 16.1	**<0.01**
DBP (mm Hg)	70 ± 11.1	65.4 ± 8.5	**<0.01**	67.5 ± 10.2	70 ± 10.5	0.18
TBF (kg)	24.5 ± 7.2	28.1 ± 11.1	**0.05**	25.8 ± 9.5	26.8 ± 7.8	0.39
TBF (%)	34.7 ± 7	35.2 ± 9.2	0.86	35.1 ± 8.2	34.3 ± 7.4	0.29
AAT (kg)	2.3 ± 0.9	2.6 ± 1.4	0.15	2.3 ± 1.2	2.8 ± 1	**<0.01**
AAT (%)	40.1 ± 9.2	39.8 ± 12.3	0.89	39.2 ± 11	42.4 ± 8.6	0.14
VAT (kg)	1.1 ± 0.7	1.1 ± 0.9	0.87	0.9 ± 0.7	1.5 ± 0.7	**<0.01**
VAT (%)	43.4 ± 17.3	38.8 ± 20.4	0.08	37.2 ± 17.8	54.9 ± 14.9	**<0.01**
SAT (kg)	1.2 ± 0.5	1.5 ± 0.8	**0.03**	1.4 ± 0.7	1.2 ± 0.6	0.11
SAT (%)	56.6 ± 17.3	61.2 ± 20.4	0.08	62.8 ± 17.8	45.1 ± 14.9	**<0.01**
VAT:SAT ratio	1.0 ± 1.0	0.9 ± 1.0	0.08	0.8 ± 0.8	1.6 ± 1.3	**<0.01**
HbA1c (mmol mol^−1^)	36.7 ± 4	34.5 ± 3.3	**<0.01**	35.0 ± 3.6	38.3 ± 3.7	**<0.01**
FPG (mmol L^−1^)	5.4 ± 0.5	5.2 ± 0.5	**0.04**	5.2 ± 0.5	5.8 ± 0.4	**<0.01**
ALT (U L^−1^)	23.8 ± 19.9	16.5 ± 10.5	**<0.01**	19.9 ± 16.7	23.4 ± 17.7	**0.03**
AST (U L^−1^)	24.7 ± 12.9	21.8 ± 5.1	0.58	23.4 ± 11.3	23.9 ± 7.4	0.2
ALP (U L^−1^)	60.9 ± 14.4	59.4 ± 14.6	0.56	60.1 ± 14.6	60.8 ± 14.1	0.74
GGT (U L^−1^)	30.6 ± 24.8	27.7 ± 27.1	0.26	26.5 ± 21.6	38.5 ± 34.8	**<0.01**
Total cholesterol (mmol L^−1^)	5.2 ± 1	5.3 ± 1.1	0.44	5.2 ± 1.1	5.1 ± 1	0.4
HDL-C (mmol L^−1^)	1.5 ± 0.4	1.7 ± 0.5	**0.05**	1.6 ± 0.4	1.4 ± 0.3	**<0.01**
TG (mmol L^−1^)	1.4 ± 0.9	1.1 ± 0.6	**0.02**	1.2 ± 0.7	1.6 ± 1	**<0.01**
LDL-C (mmol L^−1^)	3 ± 0.8	3.1 ± 0.9	0.53	3.1 ± 0.9	3 ± 0.9	0.59
Amylin (pg mL^−1^)	31.2 ± 28.9	28.7 ± 17.4	0.76	28.9 ± 25.6	34.3 ± 21.3	0.07
C-peptide (pg mL^−1^)	1206.4 ± 633.7	1257.9 ± 555.2	0.49	1186.5 ± 596.2	1359.9 ± 603.3	**0.02**
GIP (pg mL^−1^)	63.2 ± 34.5	59.9 ± 27.7	0.84	60.4 ± 32.3	66.3 ± 29.9	0.19
GLP-1 (pg mL^−1^)	201.6 ± 98.3	193.5 ± 78.5	0.62	191.1 ± 92.4	220.7 ± 80.3	**<0.01**
Glucagon (pg mL^−1^)	64.2 ± 31.3	57.7 ± 32	**0.05**	58.4 ± 31.3	71.1 ± 31.2	**<0.01**
Fasting insulin (pg mL^−1^)	758.9 ± 695.3	766.4 ± 657.5	0.88	726 ± 640.4	876.3 ± 782.1	0.2
HOMA2-IR	2.3 ± 1.6	2.3 ± 1.8	0.85	2.2 ± 1.7	2.6 ± 1.8	0.13
HOMA2-B	140.9 ± 77.1	150.7 ± 83.1	0.6	148.2 ± 81.9	135.1 ± 72	0.43

Values are reported as mean ± SD; *p*-values obtained by Kruskal–Wallis tests; *p* < 0.05 in bold. For M:F ratios, *p*-values were obtained using the chi-squared test of Independence. Abbreviations: M:F ratio (male-to-female ratio), BMI (body mass index), SBP (systolic blood pressure), DBP (diastolic blood pressure), TBF (total body fat), AAT (abdominal adipose tissue), VAT (visceral adipose tissue), SAT (subcutaneous adipose tissue), HbA1c (glycated haemoglobin A1c), FPG (fasting plasma glucose), ALT (alanine aminotransferase), AST (aspartate aminotransferase), ALP (alkaline phosphatase), GGT (γ-glutamyl transferase), TG (triacylglycerol), HDL-C (high-density lipoprotein cholesterol), LDL-C (low-density lipoprotein cholesterol), GIP (gastric inhibitory polypeptide), GLP-1 (glucagon-like peptide-1), HOMA2-IR (Homeostatic Model Assessment 2 for insulin resistance), HOMA2-B (Homeostatic Model Assessment 2 for beta-cell function).

## Data Availability

Metabolite profiling datasets (lipidomic and polar metabolites) are available from the corresponding author upon reasonable request. Host-filtered shotgun metagenomic sequence reads are available in the Sequence Read Archive under SUB15293816.

## Supplementary Materials

The following supporting information can be downloaded at: https://www.mdpi.com/article/10.3390/metabo15080522/s1, The supporting information includes Figure S1: Log fold-change of OTUs (A) and KEGG functional pathways (B) between Asian Chinese and European Caucasian participants; Figure S2: Percentage of variance explained by each omic block (polar, lipids, KEGG, OTUs) across two components of sparse multi-block PLS (sPLS) models; Figure S3: Block-specific ROC curves for ethnicity (Asian Chinese vs European Caucasian); Figure S4: Performance diagnostics for the DIABLO model built for ethnicity classification across omic blocks; Figure S5: Circos plot of cross-omic feature correlations derived from the DIABLO ethnicity model; Figure S6: Influence of glycaemic status. (A) DIABLO diagnostic plots showing multi-omics data integration according to glycaemic status. (B) Diagnostic plots visualising samples projected on the latent components, showing weak discrimination by each block (data type); Figure S7: Multivariate analysis of glycaemic status using DIABLO. Loading plots represent the top discriminating features for each dataset; Figure S8: Performance diagnostics for the DIABLO model built for glycaemic status classification across omic blocks; Figure S9: Block-specific ROC curves for glycaemic status (normoglycaemia vs. prediabetes); Figure S10: Performance metrics for sex-based discrimination PLS-DA models, across the individual omic blocks; Figure S11: Performance metrics for sex-based discrimination sPLS-DA models, across the individual omic blocks; Figure S12: Shotgun metagenomics-derived relative sequence abundance of the 10 most abundant bacterial phyla (A) and species (B), grouped by ethnicity and glycaemic status, with remaining taxa grouped under “Other”; Table S1: Multi-omic features retained across MB-sPLS models for individual clinical trait associations; Table S2: Polar metabolite features identified by DIABLO modeling; Table S3: Lipid species features identified by DIABLO modeling; Table S4: KEGG pathway features identified by DIABLO modeling; Table S5: Bacterial species (OTUs) features identified by DIABLO modeling; Table S6: Top 100 strongest correlations between lipid species and KEGG ortholog derived from the ethnicity-based DIABLO models component loadings; Table S7: Top 100 strongest correlations between Polar metabolites and KEGG ortholog derived from the ethnicity-based DIABLO models component loadings; Table S8: Top 100 strongest correlations between Polar metabolites and KEGG ortholog derived from the ethnicity-based DIABLO models component loadings; Table S9: Top 100 strongest correlations between Lipid species and OTUs derived from the ethnicity-based DIABLO models component loadings; Table S10: KEGG orthologs identified as differentially abundant.
