# Supplementary material for: Investigating Multi-Omic Signatures of Ethnicity and Dysglycaemia in Asian Chinese and European Caucasian Adults: Cross-Sectional Analysis of the TOFI_Asia Study at 4-Year Follow-Up
Source: Metabolites. 2025 Aug 1;15(8):522. doi: 10.3390/metabo15080522 (PMC12388023; doi:10.3390/metabo15080522)
Supplement: Supplementary file 1 [file metabolites-15-00522-s001.zip › Faraj_Metabolites_24062025_Supplemental material.pdf]

Supplemental materials for:

Investigating Multi-Omic Signatures of Ethnicity and Dysglycaemia in Asian Chinese and European Caucasian Adults: Cross-Sectional Analysis of the TOFI_Asia Study at 4-Year Follow-Up

Saif Faraj, Aidan Joblin-Mills, Ivana R. Sequeira-Bisson, Kok Hong Leiu, Tommy Tung, Jessica A. Wallbank, Karl Fraser, Jennifer L. Miles-Chan, Sally D. Poppitt and Michael W. Taylor

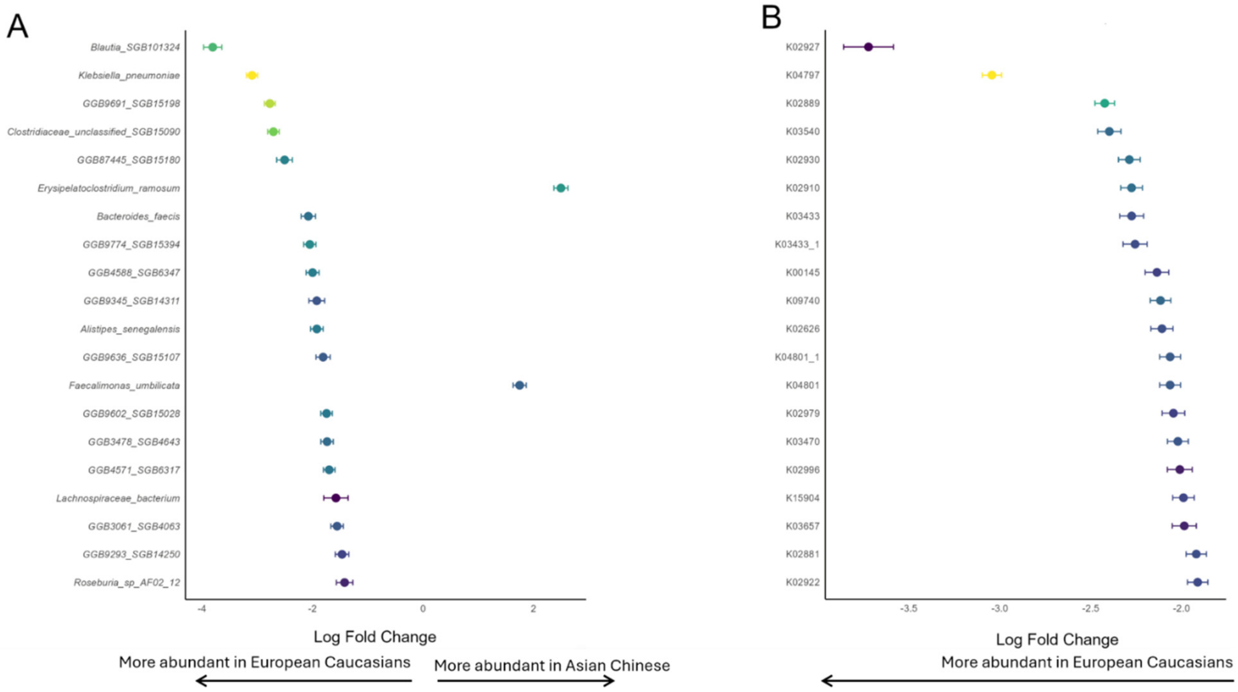

Supplementary Figure S1: Log fold-change of OTUs (A) and KEGG functional pathways (B) between Asian Chinese and European Caucasian participants. Each point represents an feature, with the horizontal axis indicating the log fold-change (higher abundance in Asian Chinese for positive values, higher abundance in European Caucasians for negative values). The colour scale corresponds to the W statistic. Only the top 20 features determined to be both differentially abundant and passing the pseudo count sensitivity analysis are displayed.

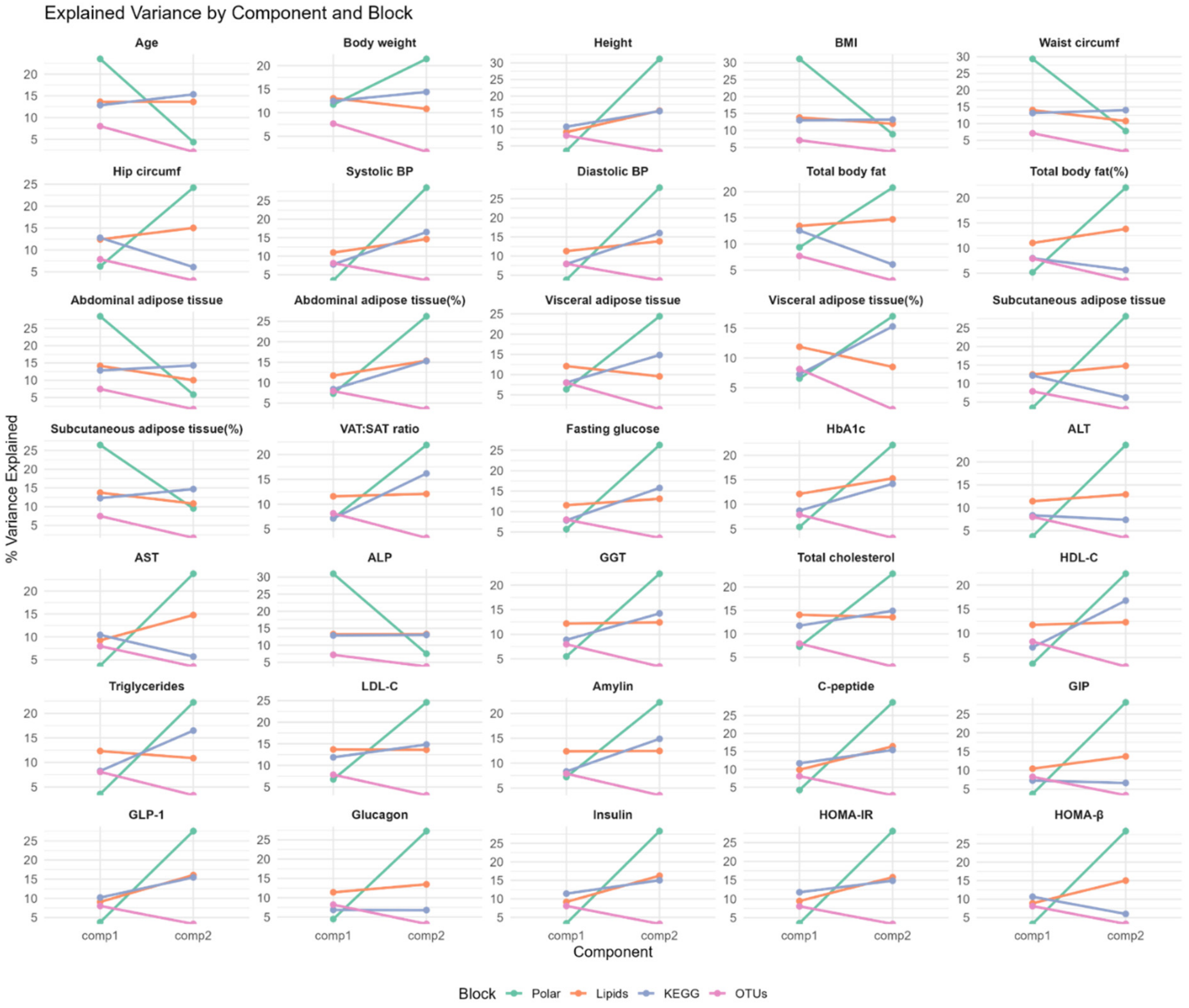

Supplementary Figure S2: Percentage of variance explained by each omic block (polar, lipids, KEGG, OTUs) across two components of sparse multi-block PLS (sPLS) models. Each facet represents a clinical trait, with variance explained by each block across components 1 and 2. Lines are colored by omic block. Models were fit independently for each trait.

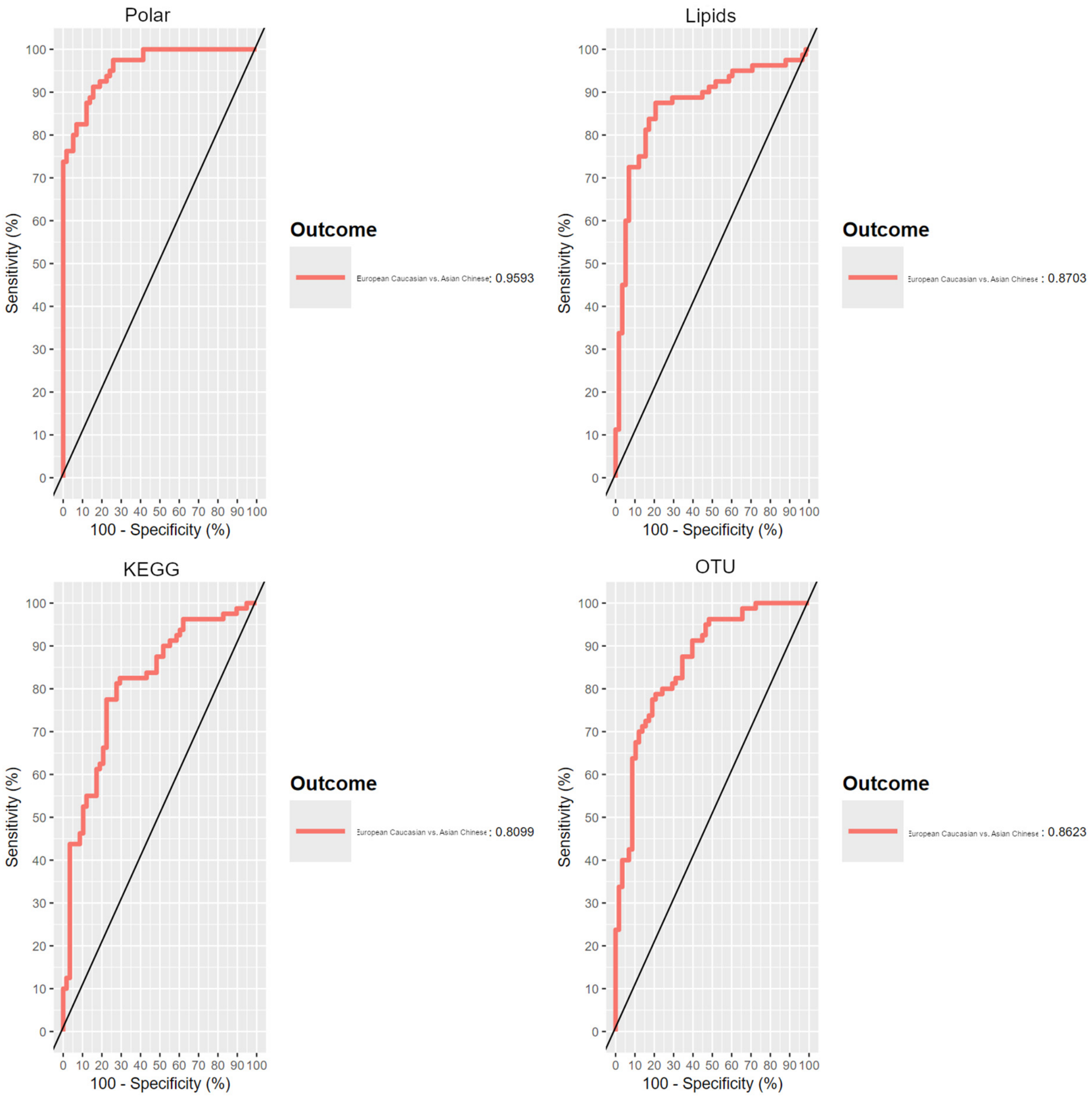

Supplementary Figure S3: Block-specific ROC curves for ethnicity (Asian Chinese vs European Caucasian). Receiver operator characteristic (ROC) curves from the DIABLO model are shown for each data block: Polar metabolites (top left), Lipids (top right), KEGG (bottom left), and OTU (bottom right). In each panel, the red line represents the DIABLO ROC curve for distinguishing between Asian Chinese and European Caucasian participants, the associated AUC (area under the curve) is printed in the legend. The Diagonal black line represents random classification.

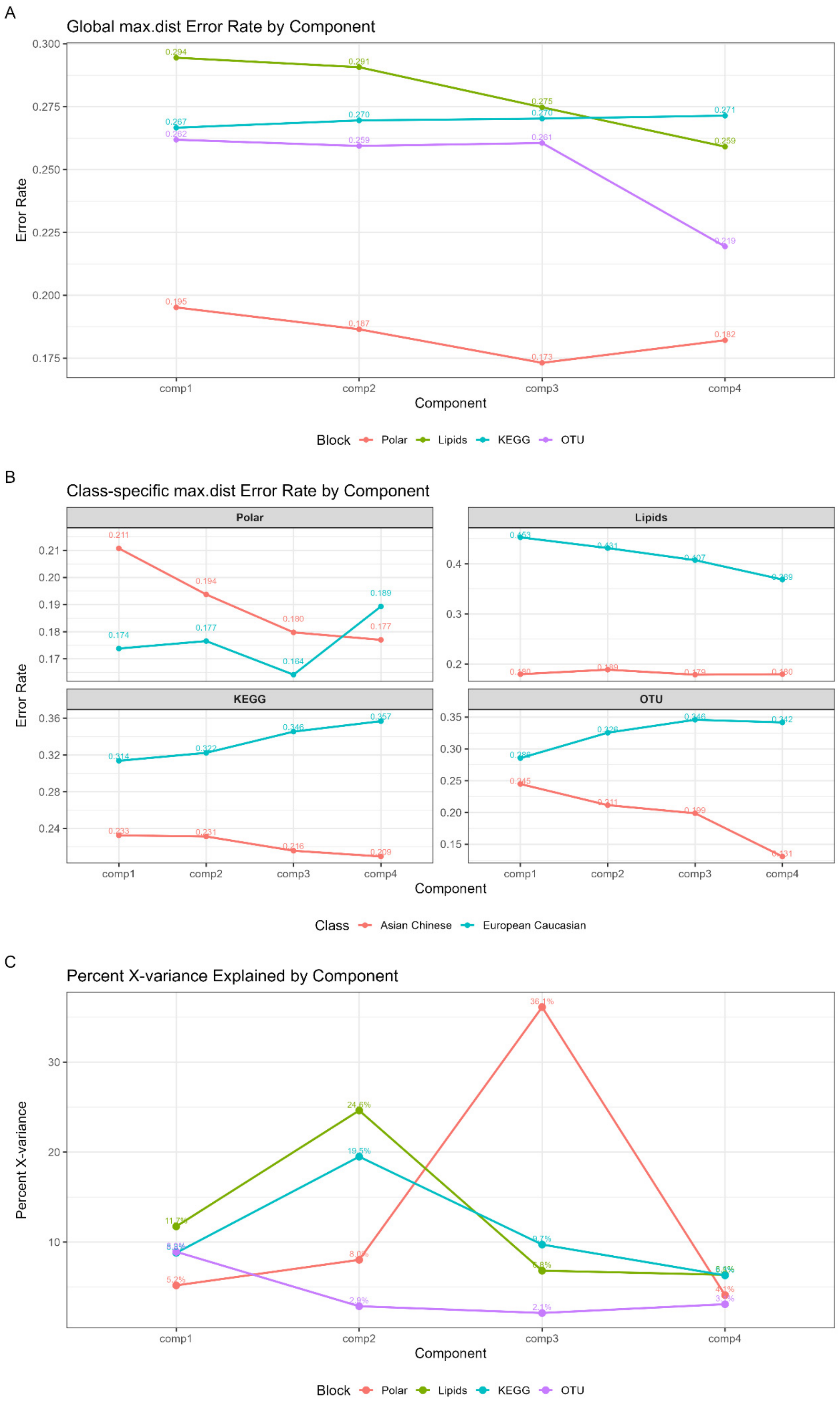

Supplementary Figure S4: Performance diagnostics for the DIABLO model built for ethnicity classification across omic blocks (A) Global balanced error rates (BER) using the maximum distance metric across 1-4 components, colored by omic block. Polar metabolites outperform other blocks, suggesting more robust discriminative performance (B). Class-specific error rates for Asian Chinese and European Caucasian participants, factored by omic block. The polar block presents the most robust error rates across components for both ethnicities. Conversely, Lipid and OUT block have higher misclassification rates, specifically amongst European Caucasians (C). Percent X- variance explained by each omic block per component. The polar block accounts for the most variance at component 3, followed by Lipid and KEGG blocks. OUT consistently explained the least variance. All together supporting the Polar block as the most informative at this ethnicity level of discrimination

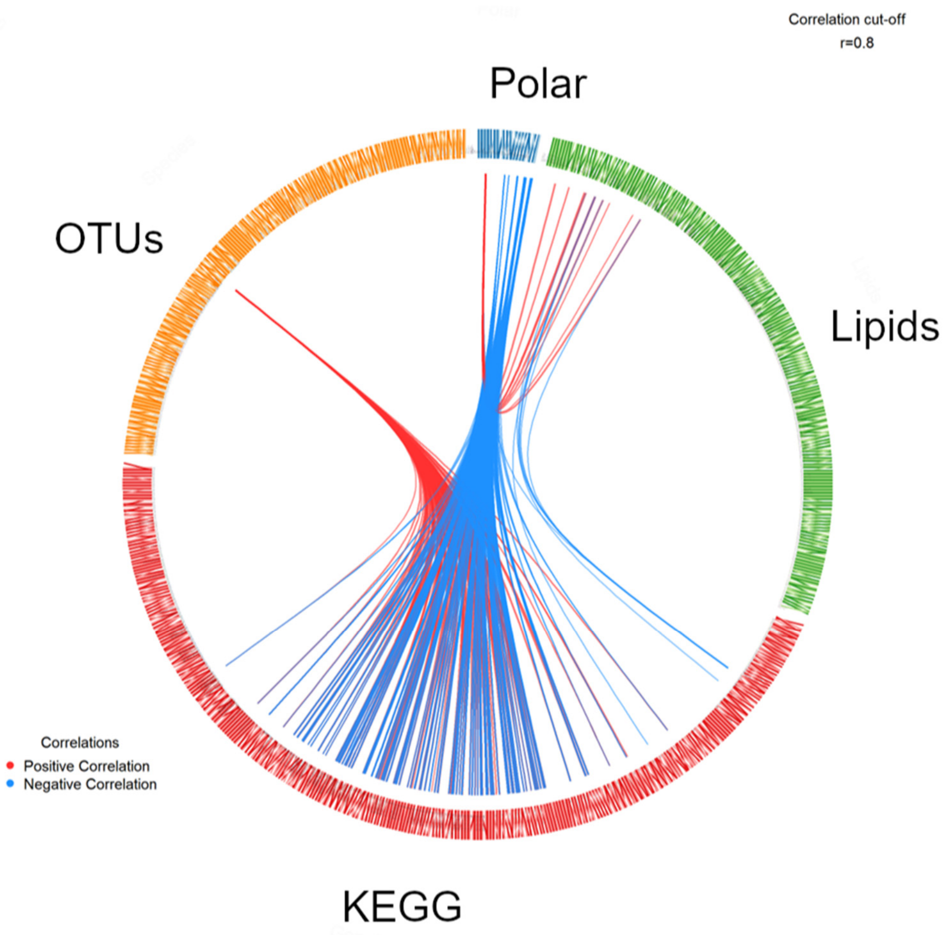

Supplementary Figure S5: Circos plot of cross-omic feature correlations derived from the DIABLO ethnicity model. Features selected by sparse multi-block PLS-DA (DIABLO) across the four omic layers (polar, blue; lipids, green; KEGG, red; OTUs, orange). The plot depicts pairwise correlations (|r| ≥ 0.8) among variables across components 1 to 4. Lines represent strong inter-omics block correlations, red correlations are positive, blue correlations are negative. Intra-block links are not displayed.

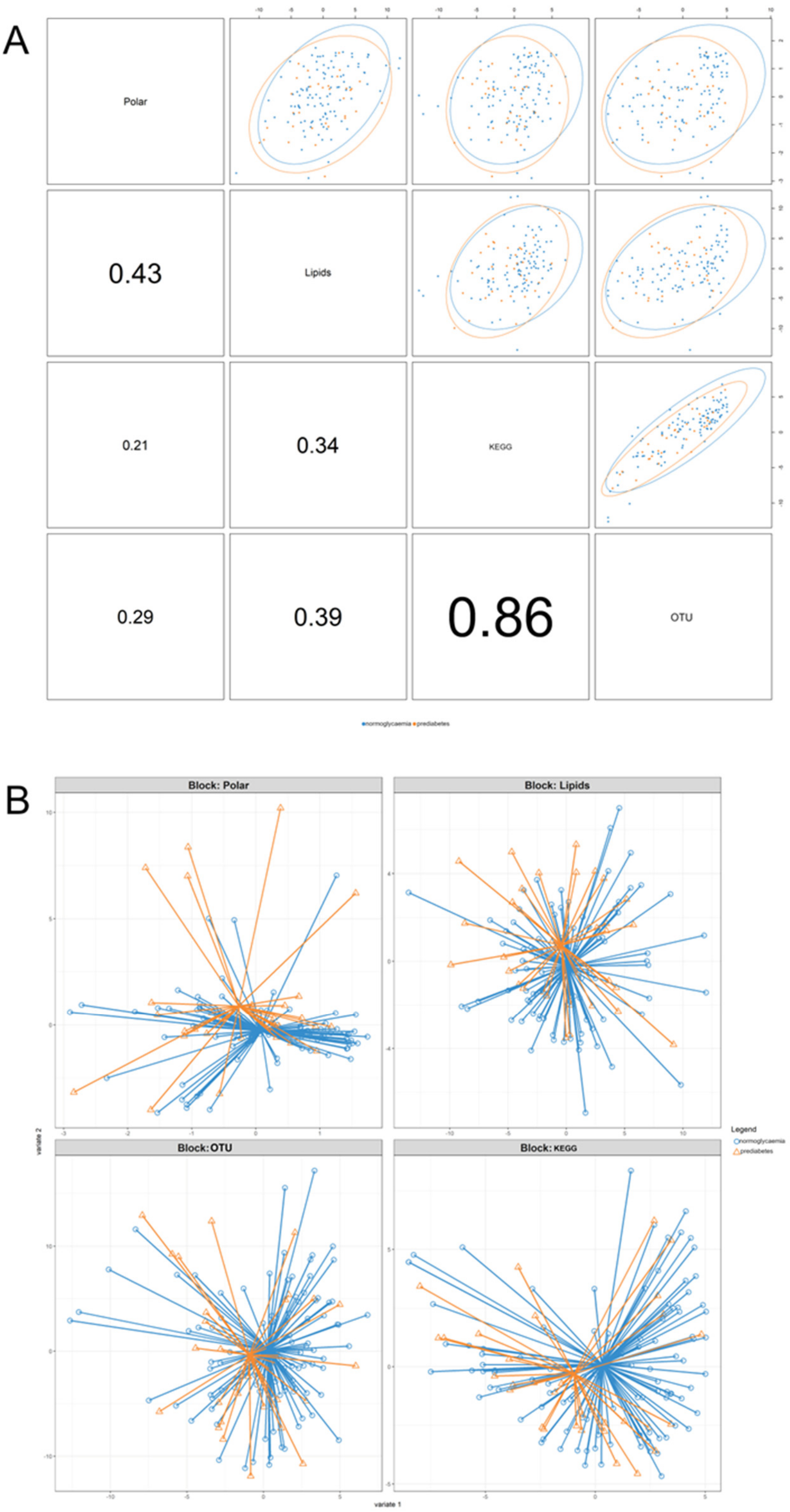

Supplementary Figure S6: Influence of glycaemic status. (A) DIABLO diagnostic plots showing multi-omics data integration according to glycaemic status, with only weak discrimination between normoglycaemia and prediabetes based on latent components from the OTU, KEGG, lipidomic, and polar metabolite datasets. The upper right of the figure contains scatter plots, coloured by group types, with ellipses representing 95% confidence. Values to the lower left represent Pearson correlation coefficients between the first components from each dataset. (B) Diagnostic plots visualising samples projected on the latent components, showing weak discrimination by each block (data type). Colours distinguish samples from normoglycaemia (blue) and prediabetes (orange) cohorts.

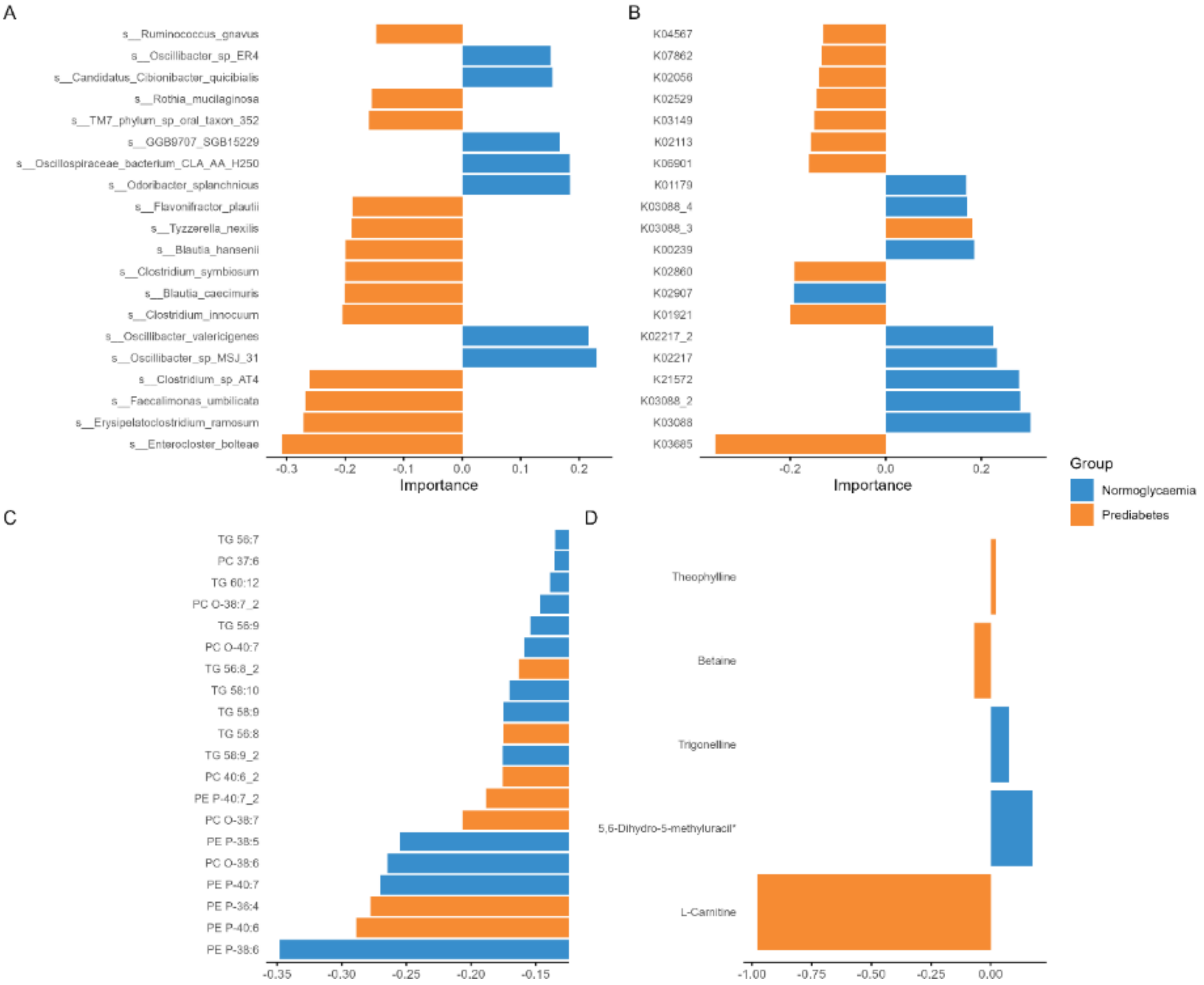

Supplementary Figure S7: Multivariate analysis of glycaemic status using DIABLO. Loading plots represent the top discriminating features for each dataset: (A) bacterial species (OTU), (B) KEGG pathways, (C) lipids, and (D) polar metabolites. Features are sorted according to discriminatory strength; the bar colour signifies that the maximal median value for a given feature is associated with either normoglycaemia (blue) or prediabetes (orange).

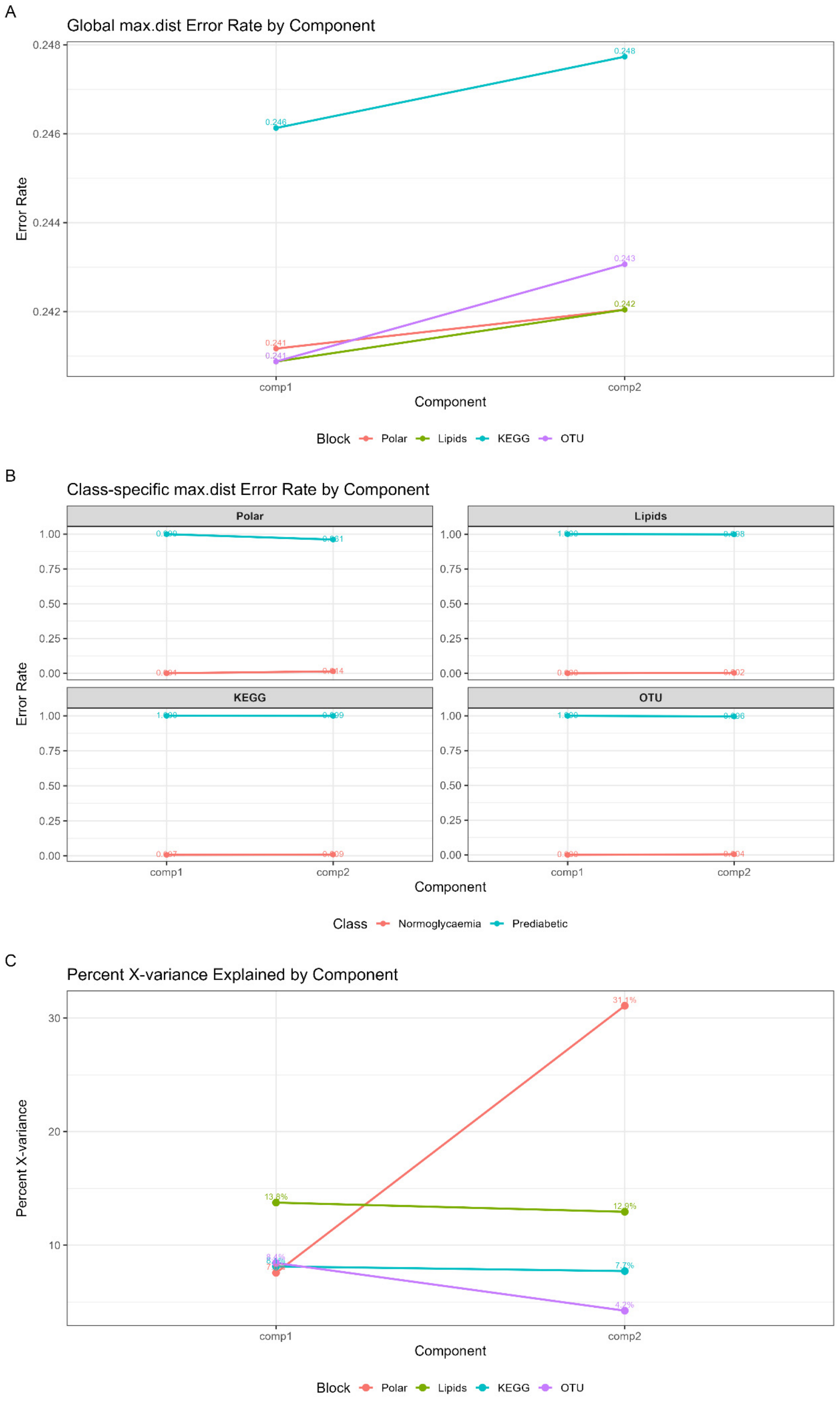

Supplementary Figure S8: Performance diagnostics for the DIABLO model built for glycaemic status classification across omic blocks (A) Global balanced error rates (BER) using the maximum distance metric across 1-4 components, colored by omic block. All blocks present similar error rates, aside from the KEGG block, which performs the poorest (B). Class-specific error rates for normoglycaemia and prediabetes participants, factored by omic block, revealing severe imbalance, with normoglycaemic participants consistently classified correctly, while prediabetic participants were universally misclassified across the omic blocks.(C) Percent X-variance explained per component for each polar block.

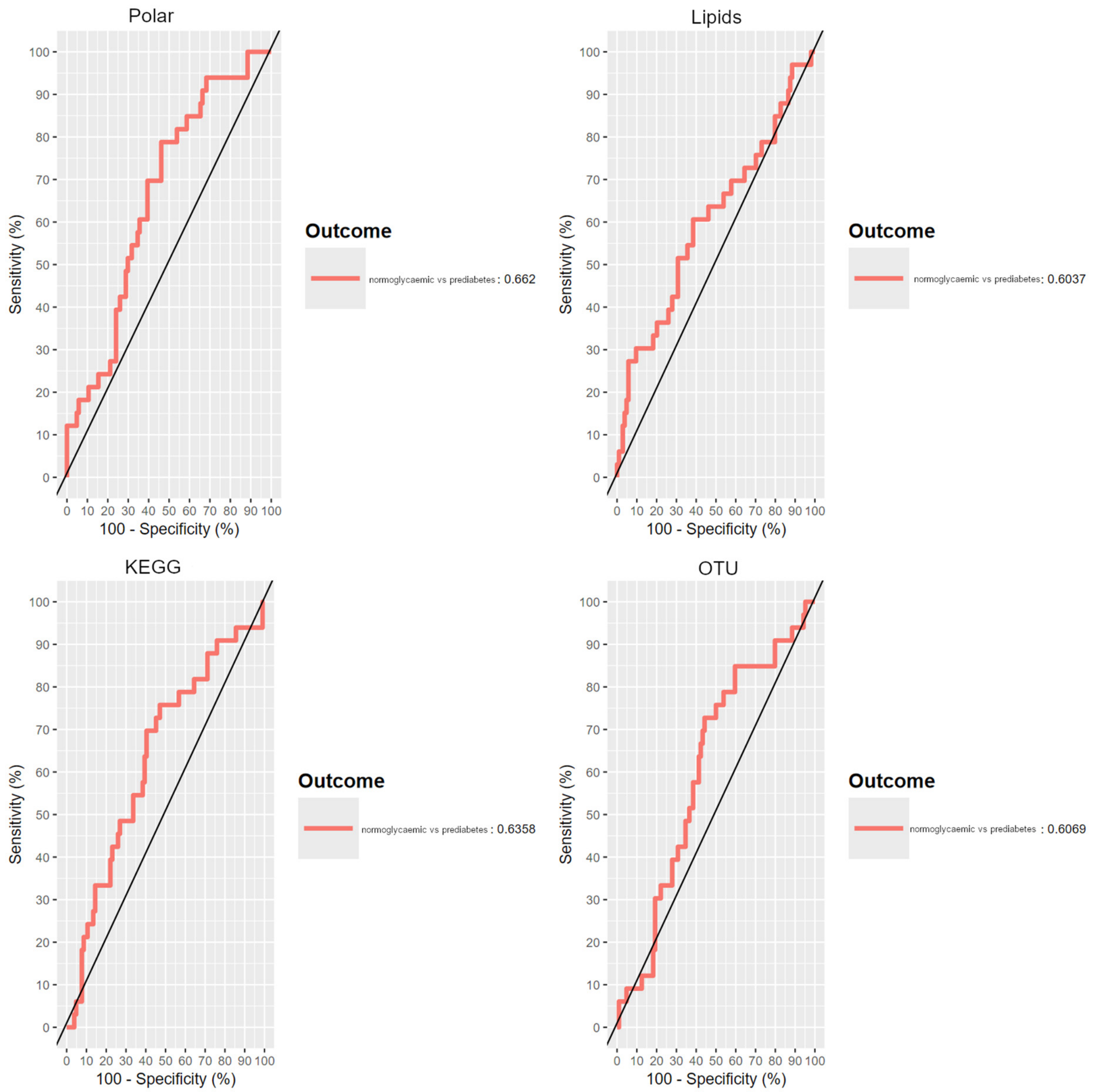

Supplementary Figure S9: Block-specific ROC curves for glycaemic status (normoglycaemia vs. prediabetes). Receiver operator characteristic (ROC) curves from the DIABLO model are shown for each data block: Polar metabolites (top left), Lipids (top right), KEGG (bottom left), and OTU (bottom right). In each panel, the red line represents the DIABLO ROC curve for distinguishing between normoglycaemia and participants with prediabetes; the associated AUC (area under the curve) is printed in the legend. The Diagonal black line represents random classification.

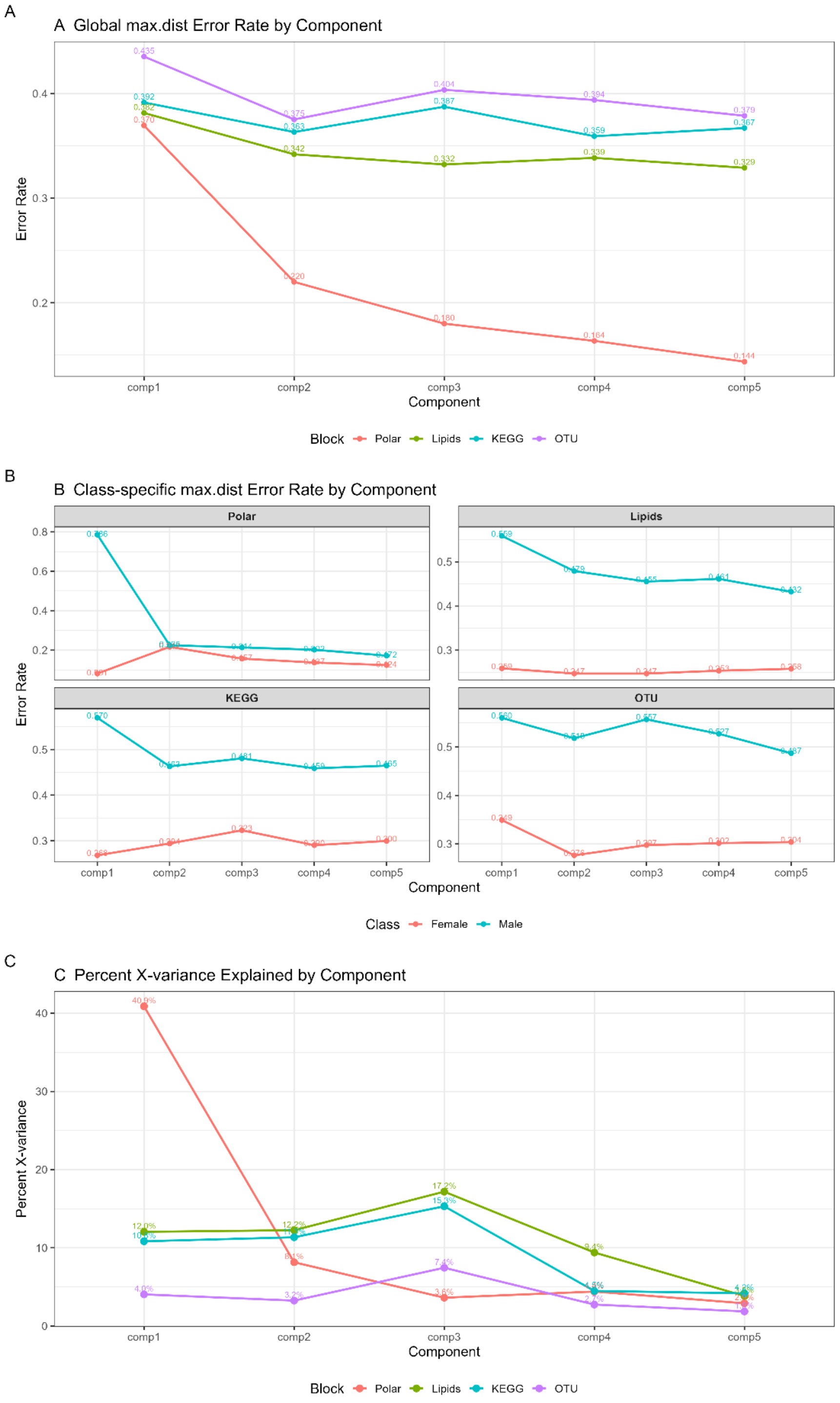

Supplementary Figure S10: Performance metrics for sex-based discrimination PLS-DA models, across the individual omic blocks. (A) Global balanced error rate (BER) across components for sPLS-DA models applied separately to each omic block (Polar, Lipids, EKGG, OTUs). Polar metabolites preformed the best, with BER decreasing across components, while microbial blocks(KEGG and OTUs) performed poorly. (B) Class specific error rates demonstrated higher misclassification rates in male participants across all omics blocks. Polar metabolites demonstrated the best classification error rates, compared to poorer classification performance in KEGG and OTU blocks. (C) Percent X-variance explained by each component revealed that polar metabolites accounted for the greatest proportion of variance in the first component.

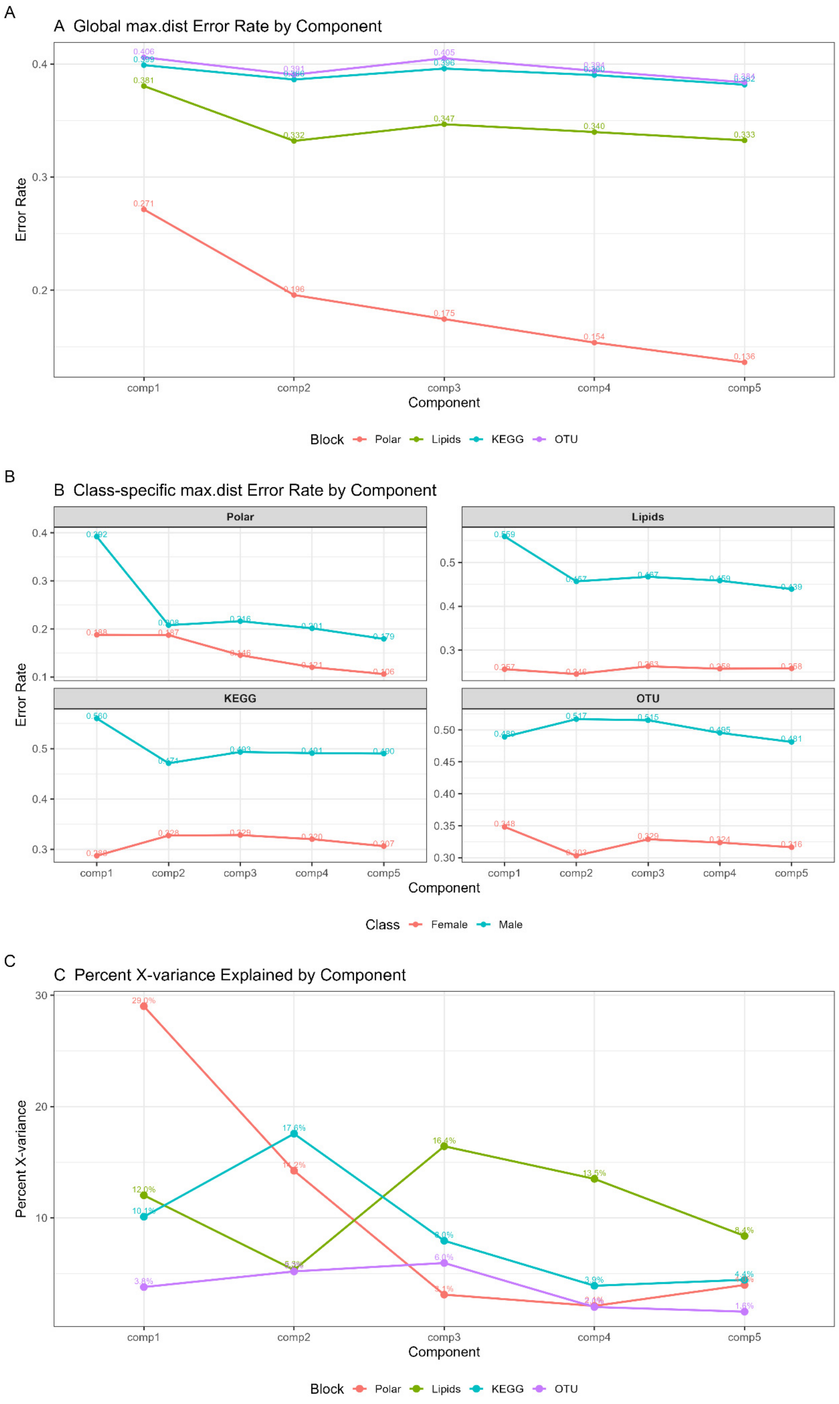

Supplementary Figure S11: Performance metrics for sex-based discrimination sPLS-DA models, across the individual omic blocks. (A) Global balanced error rate (BER) across components for sPLS-DA models applied separately to each omic block (Polar, Lipids, EKGG, OTUs). Polar metabolites preformed the best, with BER decreasing across components, while microbial blocks(KEGG and OTUs) performed poorly. (B) Class specific error rates demonstrated higher misclassification rates in male participants across all omics blocks. Polar metabolites demonstrated the best classification error rates, compared to poorer classification performance in KEGG and OTU blocks. (C) Percent X-variance explained by each component revealed that polar metabolites accounted for the greatest proportion of variance in the first component.

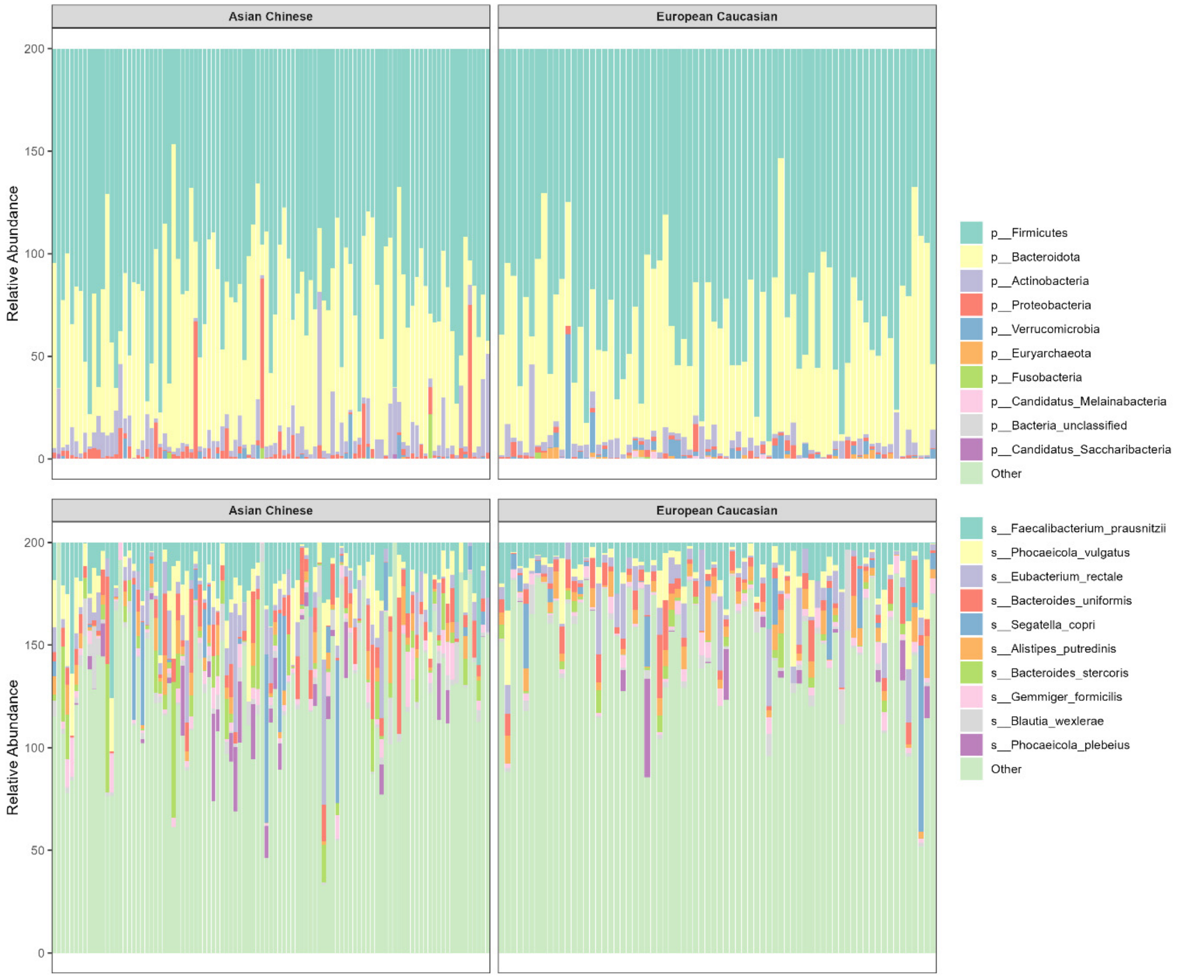

Supplementary Figure S12: Shotgun metagenomics-derived relative sequence abundance of the 10 most abundant bacterial phyla (A) and species (B), grouped by ethnicity and glycaemic status, with remaining taxa grouped under "Other". Each vertical bar corresponds to an individual participant.
